# Marine natural products as a source of novel anticancer drugs: an updated review (2019–2023)

**DOI:** 10.1007/s13659-024-00493-5

**Published:** 2025-01-24

**Authors:** Hesham R. El-Seedi, Mohamed S. Refaey, Nizar Elias, Mohamed F. El-Mallah, Faisal M. K. Albaqami, Ismail Dergaa, Ming Du, Mohamed F. Salem, Haroon Elrasheid Tahir, Maria Dagliaa, Nermeen Yosri, Hongcheng Zhang, Awg H. El-Seedi, Zhiming Guo, Shaden A. M. Khalifa

**Affiliations:** 1 Department of Chemistry, Faculty of Science, Islamic University of Madinah, 42351 Madinah, Saudi Arabia; 2 International Research Center for Food Nutrition and Safety, Jiangsu University, Zhenjiang, 212013 China; 3 International Joint Research Laboratory of Intelligent Agriculture and Agri-Products Processing, Jiangsu Education Department, Jiangsu University, Nanjing, 210024 China; 4 Department of Chemistry, Faculty of Science, Menoufia University, Shebin El-Kom, 31100107 Egypt; 5 Department of Pharmacognosy, Faculty of Pharmacy, University of Sadat City, Sadat City, 32897 Egypt; 6 Department of Laboratory Medicine, Faculty of Medicine, University of Kalamoon, P.O. Box 222, Dayr Atiyah, Syria; 7 Biology Department, Faculty of Science, Islamic University of Madinah, 42351 Madinah, Saudi Arabia; 8 Primary Health Care Corporation (PHCC), Doha, Qatar; 9 School of Food Science and Technology, National Engineering Research Center of Seafood, Dalian Polytechnic University, Dalian, 116034 China; 10 Department of Environmental Biotechnology, Genetic Engineering and Biotechnology Research Institute, GEBRI, University of Sadat City, P.O.Box:79, Sadat City, Egypt; 11 Agricultural Product Processing and Storage Lab, School of Food and Biological Engineering, Jiangsu University, Zhenjiang, 212013 Jiangsu China; 12 Department of Pharmacy, University of Napoli Federico II, Via D. Montesano 49, 80131 Naples, NA Italy; 13 School of Food and Biological Engineering, Jiangsu University, Zhenjiang, 212013 China; 14 Chemistry Department of Medicinal and Aromatic Plants, Research Institute of Medicinal and Aromatic Plants (RIMAP), Beni-Suef University, Beni-Suef, 62514 Egypt; 15 State Key Laboratory of Resource Insects, Institute of Apicultural Research, Chinese Academy of Agricultural Sciences, Beijing, 100093 China; 16 International IT College of Sweden Stockholm, Arena Academy, Hälsobrunnsgatan 6, 11361 Stockholm, Sweden; 17 Psychiatry and Neurology Department, Capio Saint Göran’s Hospital, Sankt Göransplan 1, 112 19 Stockholm, Sweden

**Keywords:** Marine natural products, Microorganism, Anticancer, Clinical trials, Drugs

## Abstract

Marine natural products have long been recognized as a vast and diverse source of bioactive compounds with potential therapeutic applications, particularly in oncology. This review provides an updated overview of the significant advances made in the discovery and development of marine-derived anticancer drugs between 2019 and 2023. With a focus on recent research findings, the review explores the rich biodiversity of marine organisms, including sponges, corals, algae, and microorganisms, which have yielded numerous compounds exhibiting promising anticancer properties. Emphasizing the multifaceted mechanisms of action, the review discusses the molecular targets and pathways targeted by these compounds, such as cell cycle regulation, apoptosis induction, angiogenesis inhibition, and modulation of signaling pathways. Additionally, the review highlights the innovative strategies employed in the isolation, structural elucidation, and chemical modification of marine natural products to enhance their potency, selectivity, and pharmacological properties. Furthermore, it addresses the challenges and opportunities associated with the development of marine-derived anticancer drugs, including issues related to supply, sustainability, synthesis, and clinical translation. Finally, the review underscores the immense potential of marine natural products as a valuable reservoir of novel anticancer agents and advocates for continued exploration and exploitation of the marine environment to address the unmet medical needs in cancer therapy

**Graphical Abstract:**

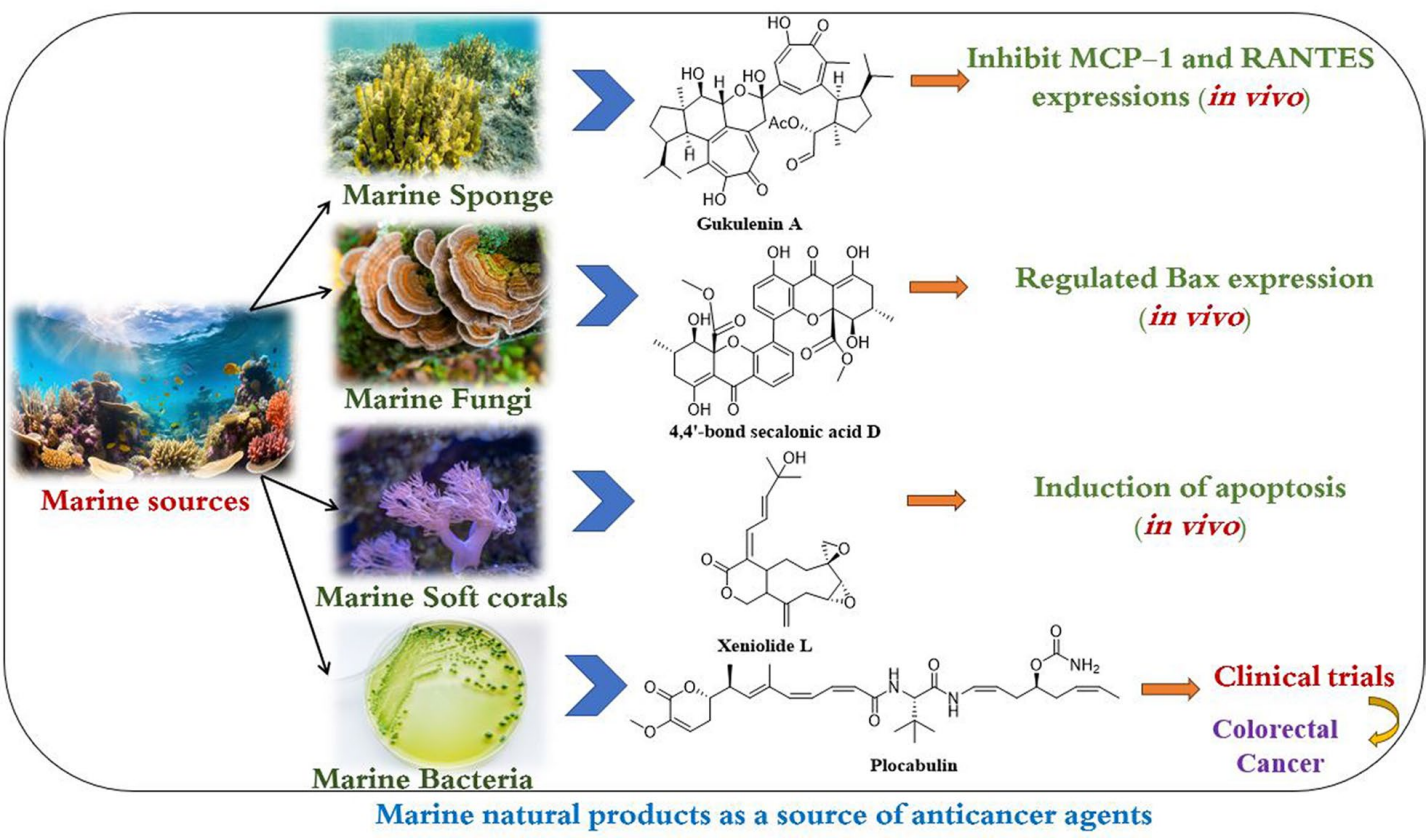

## Introduction

In the contemporary era, cancer represents a serious communal health issue and one of the main reasons for mortality worldwide, second to cardiovascular ailments [1]. Notably, tumors are not a modern ailment as previously mentioned, but it is an old one since it was mentioned in Egyptian papyrus [2]. Generally, cancer is a devastating disease that affects millions of individuals. It happens once cells in the body grow out of control, resulting in abnormal tissue growth [3]. The World Health Organization (WHO) reports that approximately one in six deaths worldwide is due to cancer, making it a significant global health issue [4]. According to recent statistics (GLOBOCAN 2020), almost 19.3 million new cancer cases with an estimated ten million cancer deaths were globally documented in 2020 [5].

**Fig. 1:**
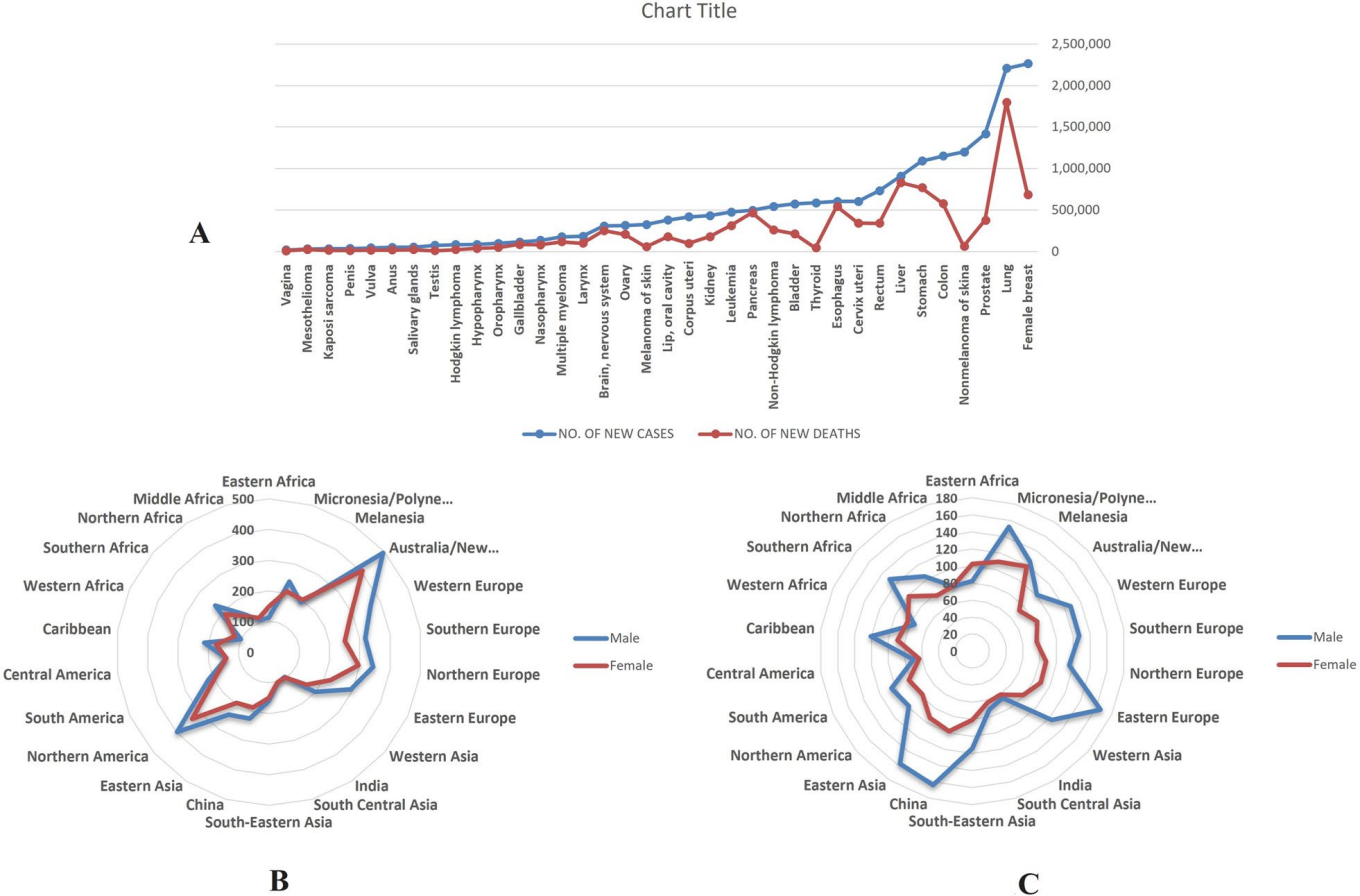
**A** New Cases and Deaths for 36 Cancers and All Cancers Combined in 2020 based on GLOBOCAN 2020; **B** Incidence Rates (Age-Standardized Rate) for 22 World Areas and Sex for All Cancers Combined in 2020 based on GLOBOCAN 2020; **C** Mortality Rates (Age-Standardized Rate) for 22 World Areas and Sex for All Cancers Combined in 2020 based on GLOBOCAN 2020

Furthermore, in 2040, the worldwide cancer encumbrance is predictable to be 28.4 million cases, a 47% growth from 2020 [5]. For instance, in 2023, 1,958,310 new cases of cancer and 609,820 cancer deaths are anticipated in the United States [6]. Figure 1A–C depicts the new cancer cases and deaths for 36 kinds of cancer in various world areas based on GLOBOCAN 2020. It is conspicuous that the lung, liver, and stomach cancers are the primary reasons for cancer mortality, where the most incidence occurs in Australia/New Zealand for both female and male sex, while the highest mortality cases belong to China and Eastern Europe countries [5].

Epidemiological studies have discussed the role of smoking, air pollution, alcohol consumption, genetic mutation, occupational exposure, viral infection, UV radiation, high obesity, junk food, and immunosuppression among others to contribute to cancer incidence [7, 8]. Despite the success of chemotherapy, radiation therapy, targeted therapy, surgery, hormone therapy, immunotherapy, and endocrine therapy in managing several types of cancer, still a plethora of patients die due to drug resistance, side effects of chemotherapy, and low immunity [9]. Thus, new approaches should be developed to combat cancer development or reduce the unwanted consequences with or without treatment [10, 11].

Marine creatures are considered one of the essential foundations of novel drugs. In this context, Carroll et al. claims that 1490 and 1425 new compounds have been discovered from marine sources in 2019 and 2021, respectively [12, 13]. Marine organisms, including algae (blue, red, green, red, and brown), microorganisms (bacteria, fungi), sponges, phytoplankton (dinoflagellates), mollusks (sea cucumbers and hares), coelenterates (sea anemones, gorgonians, and soft corals), and bryozoans, have long been known to produce a wide variety of secondary metabolites with diverse and complex chemical structures [14, 15]. Numerous of these compounds have been found to exhibit remarkable biological potential, including anticancer, antimicrobial, and anti-inflammatory properties [13]. Importantly, marine-derived compounds have shown promise as potent inhibitors of cancer cell growth and have demonstrated activity toward a wide spectrum of cancer types, among them some that are resistant to conventional chemotherapy [14]. These compounds offer a unique and largely untapped source of chemical diversity that can be exploited in the search for new and effective cancer treatments [16]. This has led to a growing body of research focused on the detection and improvement of marine-derived anticancer drugs, with many compounds undergoing preclinical and clinical evaluation.

The potential for discovery of new anticancer medicines from marine natural ingredients has attracted increasing attention in recent years. Thus, the main goal of the present manuscript is to screen the updated and latest investigation on marine natural products with anticancer activity as a continuation of our previously published review in 2019 [17]. The review will cover their action mechanism, pharmacological properties, and potential therapeutic applications. As well as the review highlights the challenges and opportunities associated with the detection and improvement of marine-derived anticancer drugs, and finally it discusses the prospects for future research in this exciting and rapidly evolving field.

## Methodology

An extensive survey of the chemical compounds and anticancer activities of marine natural products was conducted in scientific databases, including Google Scholar and SciFinder. In the present review, search terms “marine algae’’, “marine soft corals’’, “microalgae’’, “marine bacteria’’, “marine fungi’’ were used either to search “in vivo’’ or “in vitro studies against cancer’’. Also, “clinical trials of marine against cancer’’ and “marketed marine drugs against cancer’’ were used for data collection. In total, 88 publications were included from the year 2019 to December 2023. From those, 61 studies were used to analyze recent updates and information related to marine anticancer compounds in the context of the topics mentioned above.

## Different marine sources against cancer.

### Secondary metabolites of marine sponges against cancer.

#### In vitro studies of bioactive compounds from marine sponge.

**Fig. 2:**
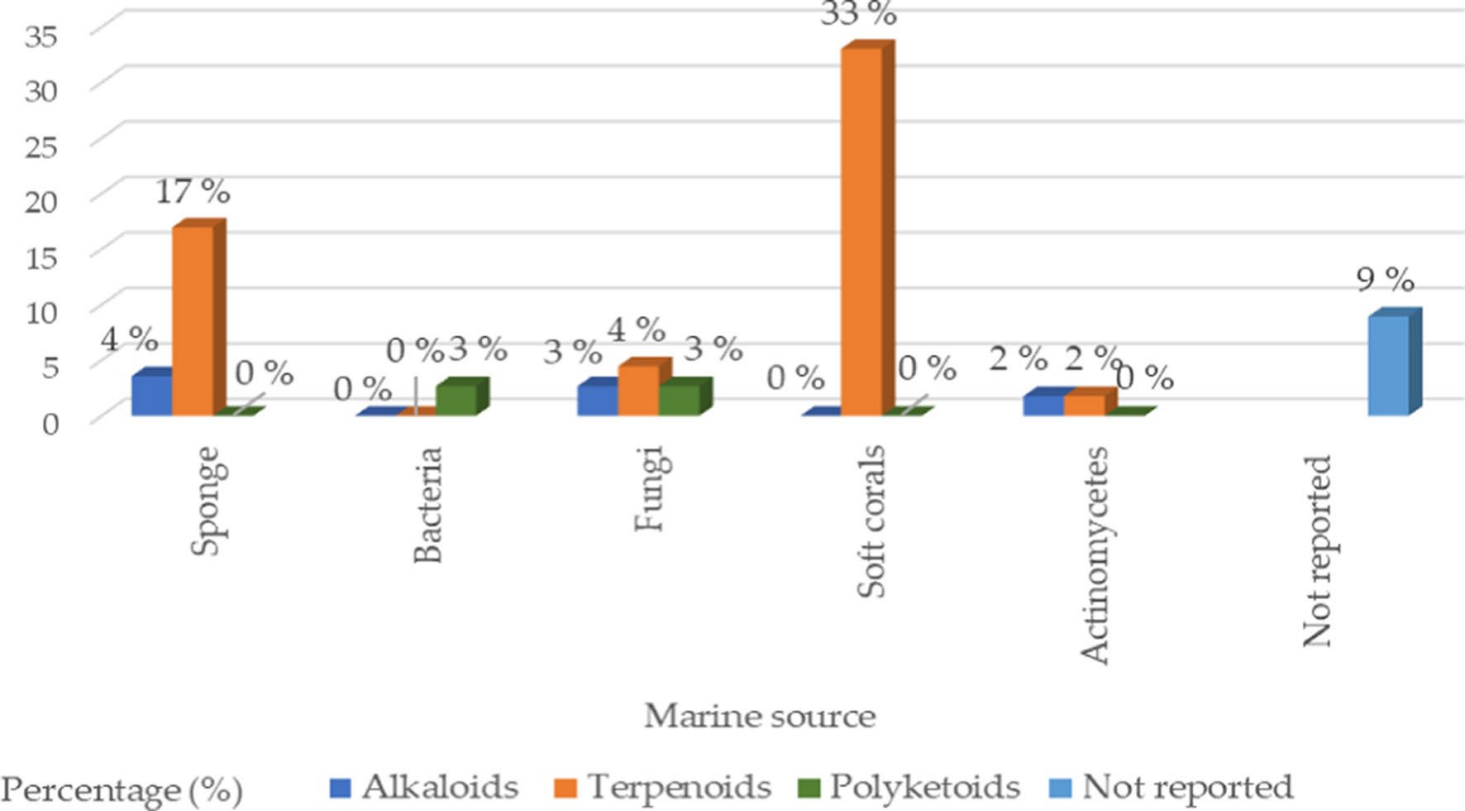
Flowchart of the screened classes from different sources with the anticancer impact

**Fig. 3:**
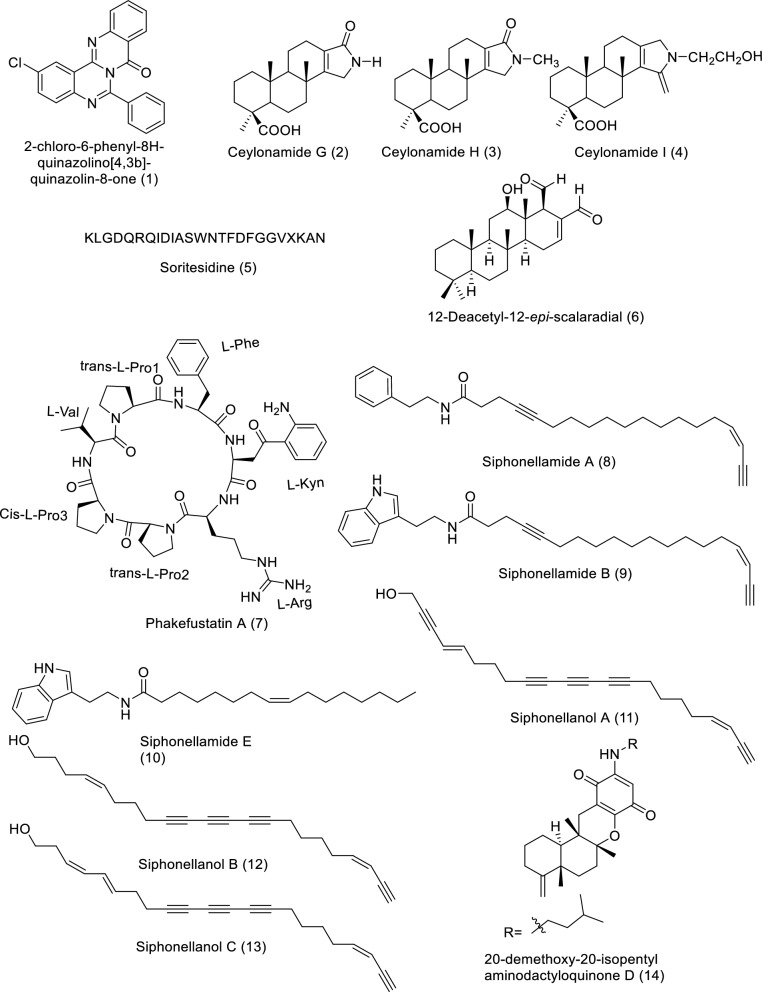

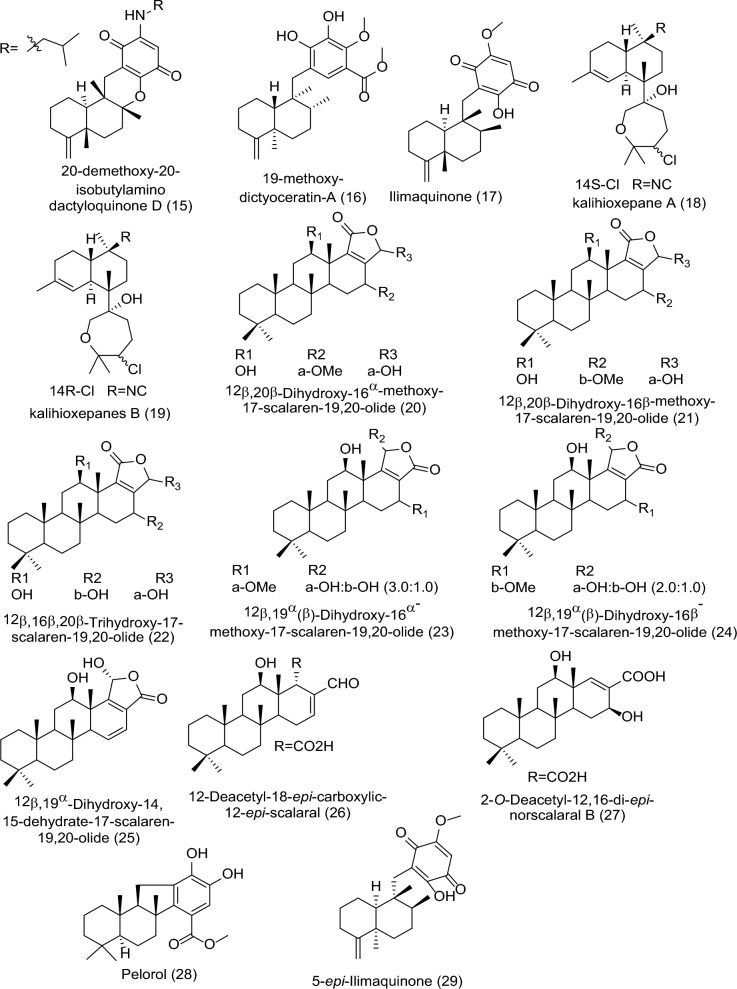
Structures of bioactive compounds isolated from marine sponge between 2019 and 2023 with in vitro studies

**Fig. 4:**
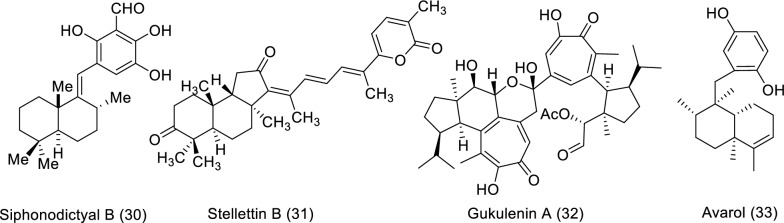
Structures of bioactive compounds isolated from marine sponge between 2019 and 2023 with in vivo studies

The marine sources are rich in different classes of secondary metabolites with various pharmacological targets as shown in Fig. 2. The sedentary lifestyle of marine sponges allows them to produce various bioactive compounds to protect themselves from predators. These bioactive compounds have a variety of medical applications, *i.e.,* cancer treatment [18–20]. Cytarabine, fludarabine phosphate, nelarabine, and eribulin mesylate are the four molecules produced by marine sponges (or by symbiotic cyanobacteria) that have received Food and Drug Administration (FDA) approval as anti-tumor medications. FDA and European Medicines Agency (EMA) later authorized fludarabine phosphate and nelarabine as anticancer drugs for leukemia and lymphoma [21, 22]. Herein we survey the different isolated bioactive compounds derived from marine sponge and involved in preclinical and clinical anticancer studies between 2019 and 2023 as illustrated in Tables 1, 2, and Figs. 3 and 4. It is significant to notice that we detailed the highly bioactive compounds in more depth.

**Table 1:** In vitro studies of bioactive compounds isolated from marine sponge between 2019 and 2023

Compound Name	Class of compound	Marine Source	Type of Cancer	Pharmacological effects	Mechanism	References
2-Chloro-6-phenyl-8*H*-quinazolino [4,3b]quinazolin-8-one (**1**)	Quinazoline derivative	*Hyrtios erectus*, Sponge	Breast cancer	Model: MCF-7 Assay: MTT assay IC_50_ for 24: 22.67 ± 1.53 µg/mL IC_50_ for 48: 13.04 ± 1.03 µg/mL Positive control: Cyclophosphamide IC_50_ for 24: 15.11 ± 1.16 µg/mL IC_50_ for 48: 8.11 ± 0.84 µg/mL	Apoptosis pathways (extrinsic or intrinsic) and reactive oxygen species (ROS) production	[23]
Ceylonamide G (**2**)	Diterpene alkaloid	*Spongia* sp. Sponge	Prostate cancer	Model: DU145 Assay: two-dimensional monolayer culture and spheroid of three-dimensional cell culture IC_50_ for 2D culture: 6.9 μM PC: Taxol IC_50_: 2.6 nM MEC for spheroid: 10 μM PC: Taxol MEC: 10 nM	Not reported	[24]
Ceylonamide H (**3**)	Diterpene alkaloid	*Spongia* sp. Sponge	Prostate cancer	Model: DU145 Assay: two-dimensional monolayer culture and spheroid of three-dimensional cell culture IC_50_ for 2D culture: > 100 μM PC: Taxol IC_50_: 2.6 nM MEC for spheroid: > 100 μM PC: Taxol MEC: 10 nM	Not reported	[24]
Ceylonamide I (**4**)	Diterpene alkaloid	*Spongia* sp. Sponge	Prostate cancer	Model: DU145 Assay: two-dimensional monolayer culture and spheroid of three-dimensional cell culture IC_50_ for 2D culture: > 100 μM PC: Taxol IC_50_: 2.6 nM MEC for spheroid: > 100 μM PC: Taxol MEC: 10 nM	Not reported	[24]
Soritesidine (**5**)	Protein	*Spongosorites* sp. Sponge	Cervical cancer and leukemia cancer	Model: HeLa and L1210 Assay: NR IC_50_ for HeLa: 0.062 ng/mL IC_50_ for L1210: 12.11 ng/mL	Not reported	[25]
12-Deacetyl-12-*epi*-scalaradial (**6**)	Scalarane sesterterpenoid	*Hippospongia* sp. Sponge	Cervical cancer	Model: HeLa cells Assay: MTT assay and Western blotting Concentration: 30 µM IC_50_: 13.74 µM	Mediating the apoptosis pathway and suppression mitogen-activated protein kinase/extracellular signal-regulated kinase (MAPK/ERK)	[31]
Phakefustatin A (**7**)	Cycloheptapeptide	*Phakellia fusca*/Sponge	Cervical cancer breast cancer, colon cancer, lung cancer, liver cancer, and nonmalignant cells (H9c2 and HEK293T)	Model: MCF-7, HeLa, NCI-H460, PC9, SW480, HepG2, H9c2 and HEK293T Assay: NR IC_50_ for MCF-7: 3.4 ± 1.2 μM PC: cisplatin IC_50_: 4.4 ± 0.3 μM IC_50_ for HeLa: 6.2 ± 0.3 μM PC: cisplatin IC_50_: 4.8 ± 0.8 μM IC_50_ for NCI-H460: 7.1 ± 0.6 μM PC: cisplatin IC_50_: 3.2 ± 1.1 μM IC_50_ for PC9: 12 ± 0.4 μM PC: cisplatin IC_50_: 3.9 ± 0.1 μM IC_50_ for SW480: > 40 μM PC: cisplatin IC_50_: 3.8 ± 0.3 μM IC_50_ for HepG2: > 40 μM PC: cisplatin IC_50_: 4.2 ± 0.5 μM IC_50_ for H9c2: > 100 μM PC: cisplatin IC_50_: 7.9 ± 0.7 μM IC_50_ for HEK293T: > 100 μM PC: cisplatin IC_50_: NR	Apoptosis and cell growth inhibition depending on the signaling pathway RXRα-mediated PI3K/Akt	[26]
Siphonellamide A (**8**)	Polyacetylene amides	*Siphonochalina siphonella*/Sponge	Breast cancer, cervical cancer, and lung cancer	Model: HeLa, MCF-7 and A549 Assay: MTT assay IC_50_ for HeLa: 9.4 μM PC: 5-Fluorouracil IC_50_: 28.4 μM IC_50_ for MCF-7: 18.0 μM PC: 5-Fluorouracil IC_50_: 34.7 μM IC_50_ for A549: 24.2 μM PC: 5-Fluorouracil IC_50_: 14.6 μM	The acetylene functionality may be responsible for their cytotoxic properties	[27]
Siphonellamide B (**9**)	Polyacetylene amides	*Siphonochalina siphonella*/Sponge	Breast cancer, cervical cancer, and lung cancer	Model: HeLa, MCF-7 and A549 Assay: MTT assay IC_50_ for HeLa: 17.4 μM PC: 5-Fluorouracil IC_50_: 28.4 μM IC_50_ for MCF-7: 34.1 μM PC: 5-Fluorouracil IC_50_: 34.7 μM IC_50_ for A549: 25.9 μM PC: 5-Fluorouracil IC_50_: 14.6 μM	The acetylene functionality may be responsible for their cytotoxic properties	[27]
Siphonellamide E (**10**)	Fatty amide	*Siphonochalina siphonella*/Sponge	Breast cancer, cervical cancer, and lung cancer	Model: HeLa, MCF-7 and A549 Assay: MTT assay IC_50_ for HeLa: 78.4 μM PC: 5-Fluorouracil IC_50_: 28.4 μM IC_50_ for MCF-7: > 100 μM PC: 5-Fluorouracil IC_50_: 34.7 μM IC_50_ for A549: > 100 μM PC: 5-Fluorouracil IC_50_: 14.6 μM	The acetylene functionality may be responsible for their cytotoxic properties	[27]
Siphonellanol A (**11**)	Polyacetylenic alcohol	*Siphonochalina siphonella*/Sponge	Cervical cancer, breast cancer and lung cancer	Model: HeLa, MCF-7 and A549 Assay: MTT assay IC_50_ for HeLa: 26.5 μM PC: 5-Fluorouracil IC_50_: 28.4 μM IC_50_ for MCF-7: 54.9 μM PC: 5-Fluorouracil IC_50_: 34.7 μM IC_50_ for A549: 59.8 μM PC: 5-Fluorouracil IC_50_: 14.6 μM	Not reported	[32]
Siphonellanol B (**12**)	Polyacetylenic alcohol	*Siphonochalina siphonella*/Sponge	Cervical cancer, breast cancer and lung cancer	Model: HeLa, MCF-7 and A549 Assay: MTT assay IC_50_ for HeLa: 26.2 μM PC: 5-Fluorouracil IC_50_: 28.4 μM IC_50_ for MCF-7: 69.2 μM PC: 5-Fluorouracil IC_50_: 34.7 μM IC_50_ for A549: 59.9 μM PC: 5-Fluorouracil IC_50_: 14.6 μM	Not reported	[32]
Siphonellanol C (**13**)	Polyacetylenic alcohol	*Siphonochalina siphonella*/Sponge	Cervical cancer, breast cancer and lung cancer	Model: HeLa, MCF-7 and A549 Assay: MTT assay IC_50_ for HeLa: 25.9 μM PC: 5-Fluorouracil IC_50_: 28.4 μM IC_50_ for MCF-7: 57.6 μM PC: 5-Fluorouracil IC_50_: 34.7 μM IC_50_ for A549: 58.5 μM PC: 5-Fluorouracil IC_50_: 14.6 μM	Not reported	[32]
20-Demethoxy-20-isopentylami-nodactyloquinone D (**14**)	Sesquiterpene quinone	*Dactylospongia elegans*, Sponge	Pancreatic cancer, prostate cancer, and liver cancer	Model: SW1990, DU145, PANC-1 and Huh7 Assay: NR IC_50_ for DU145, SW1990, Huh7, and PANC-1: > 50 µM PC: Cisplatin IC_50_: 2.9 µM, 1.2 µM, 2.2 µM and 4.6 µM; respectively	Not reported	[33]
20-Demethoxy-20-isobutyla-minodactyloquinone D (**15**)	Sesquiterpene quinone	*Dactylospongia elegans*, Sponge	Liver cancer, prostate cancer and pancreatic	Model: SW1990, DU145, PANC-1 and Huh7 Assay: NR IC_50_ for DU145, SW1990, Huh7, and PANC-1: > 50 µM PC: Cisplatin IC_50_: 2.9 µM, 1.2 µM, 2.2 µM and 4.6 µM respectively	Not reported	[33]
19-Methoxy-dictyoceratin A (**16**)	Sesquiterpene quinone	*Dactylospongia elegans*, Sponge	Liver cancer, prostate cancer and pancreatic	Model: SW1990, DU145, PANC-1 and Huh7 Assay: NR IC_50_ for DU145: 24.4 µM PC: Cisplatin IC_50_: 2.9 µM IC_50_ for SW1990: 21.4 µM PC: Cisplatin IC_50_: 1.2 µM IC_50_ for Huh7: 17.4 µM PC: Cisplatin IC_50_: 2.2 µM IC_50_ for PANC-1: 37.8 µM PC: Cisplatin IC_50_: 4.6 µM	Not reported	[33]
Ilimaquinone (**17**)	Sesquiterpene quinine	*Hippospongia metachromia*, Sponge	Colorectal cancer	Model: HCT-116 Assay: MTT assay IC_50_: 17.89 μM PC: NR	Ilimaquinone trigger mitochondria-mediated apoptosis through the decrease in mitochondrial membrane potential and activate caspase-9/-3, DNA damage, and a reduction in B cell lymphoma-2 (Bcl-2) proportion	[28]
Kalihioxepane A (**18**)	Diterpenoid	*Acanthella cavernosa*/Sponge	Erythroleukemic cancer, pancreatic cancer, chemoresistant lung cancer, chemosensitive lung cancer and breast cancer	Model: K562, ASPC-1, H69AR, H69 and MDA-MB-231 Assay: MTT assay and SRB method IC_50_ for K562: 6.57 μmol/L PC: Doxorubicin IC_50_: 0.252 μmol/L IC_50_ for ASPC-1: 16.17 μmol/L PC: Doxorubicin IC_50_: 0.023 μmol/L IC_50_ for H69AR: 21.85 μmol/L PC: Doxorubicin IC_50_: 15.120 μmol/L IC_50_ for H69: 3.60 μmol/L PC: Doxorubicin IC_50_: 0.980 μmol/L IC_50_ for MDA-MB-231: > 30 μmol/L PC: Doxorubicin IC_50_: 0.176 μmol/L	Isocyano substituent was important for cytotoxicity	[29]
Kalihioxepane B (**19**)	Diterpenoid	*Acanthella cavernosa*/Sponge	Erythroleukemic cancer, pancreatic cancer, chemoresistant lung cancer, chemosensitive lung cancer and breast cancer	Model: K562, ASPC-1, H69AR, H69 and MDA-MB-231 Assay: MTT assay and SRB method IC_50_ for K562: 8.73 μmol/L PC: Doxorubicin IC_50_: 0.252 μmol/L IC_50_ for ASPC-1: > 30 μmol/L PC: Doxorubicin IC_50_: 0.023 μmol/L IC_50_ for H69AR: > 30 μmol/L PC: Doxorubicin IC_50_: 15.120 μmol/L IC_50_ for H69: > 30 μmol/L PC: Doxorubicin IC_50_: 0.980 μmol/L IC_50_ for MDA-MB-231: > 30 μmol/L PC: Doxorubicin IC_50_: 0.176 μmol/L	Isocyano substituent was important for cytotoxicity	[29]
12β,20β-Dihydroxy-16α-methoxy-17-scalaren-19,20-olide (**20**)	Sesterterpenoid	*Hyrtios erectus*/Sponge	Breast cancer and Cervical cancer	Model: HeLa and MCF-7 Assay: MTS assay IC_50_ for HeLa: 53.4 μM PC: Staurosporine IC_50_: 0.18 μM IC_50_ for MCF-7: 27.3 μM PC: staurosporine IC_50_: 0.13 μM	NR	[34]
12β,20β-Dihydroxy-16β-methoxy-17-scalaren-19,20-olide (**21**)	Sesterterpenoid	*Hyrtios erectus*/Sponge	Breast cancer and Cervical cancer	Model: HeLa and MCF-7 Assay: MTS assay IC_50_ for HeLa: 46.2 μM PC: staurosporine IC_50_: 0.18 μM IC_50_ for MCF-7: 26.2 μM PC: staurosporine IC_50_: 0.13 μM	NR	[34]
12β,16β,20β-Trihydroxy-17-scalaren-19,20-olide (**22**)	Sesterterpenoid	*Hyrtios erectus*/Sponge	Breast cancer and Cervical cancer	Model: HeLa and MCF-7 Assay: MTS assay IC_50_ for HeLa: 60.1 μM PC: staurosporine IC_50_: 0.18 μM IC_50_ for MCF-7: 29.9 μM PC: staurosporine IC_50_: 0.13 μM	NR	[34]
12β,19α(β)-Dihydroxy-16α-methoxy-17-scalaren-19,20-olide (**23**)	Sesterterpenoid	*Hyrtios erectus*/Sponge	Breast cancer and Cervical cancer	Model: HeLa and MCF-7 Assay: MTS assay IC_50_ for HeLa: 61.3 μM PC: staurosporine IC_50_: 0.18 μM IC_50_ for MCF-7: 45.9 μM PC: staurosporine IC_50_: 0.13 μM	NR	[34]
12β,19α(β)-Dihydroxy-16β-methoxy-17-scalaren-19,20-olide (**24**)	Sesterterpenoid	*Hyrtios erectus*/Sponge	Breast cancer and Cervical cancer	Model: HeLa and MCF-7 Assay: MTS assay IC_50_ for HeLa: 70.7 μM PC: staurosporine IC_50_: 0.18 μM IC_50_ for MCF-7: 76.4 μM PC: staurosporine IC_50_: 0.13 μM	NR	[34]
12β,19α-Dihydroxy-14,15-dehydrate-17-scalaren-19,20-olide (**25**)	Sesterterpenoid	*Hyrtios erectus*/Sponge	Breast cancer and Cervical cancer	Model: HeLa and MCF-7 Assay: MTS assay IC_50_ for HeLa: 59.3 μM PC: staurosporine IC_50_: 0.18 μM IC_50_ for MCF-7: 33.8 μM PC: staurosporine IC_50_: 0.13 μM	NR	[34]
12-Deacetyl-18-*epi*-carboxylic-12-*ep*i-scalaral (**26**)	Sesterterpenoids	*Hyrtios erectus*/Sponge	Breast cancer and Cervical cancer	Model: HeLa and MCF-7 Assay: MTS assay IC_50_ for HeLa: > 80.0 μM PC: staurosporine IC_50_: 0.18 μM IC_50_ for MCF-7: > 80.0 μM PC: staurosporine IC_50_: 0.13 μM	NR	[34]
2-*O*-Deacetyl-12,16-di-*epi*-norscalaral B (**27**)	Sesterterpenoid	*Hyrtios erectus*/Sponge	Breast cancer and Cervical cancer	Model: HeLa and MCF-7 Assay: MTS assay IC_50_ for HeLa: > 80.0 μM PC: staurosporine IC_50_: 0.18 μM IC_50_ for MCF-7: > 80.0 μM PC: staurosporine IC_50_: 0.13 μM	NR	[34]
Pelorol (**28**)	Labdane-type diterpene	*Dactylospongia elegans*/Sponge	Melanoma cancer	Model: 501Mel Assay: Cell viability assay IC_50_ for 24 h: 12.51 ± 1.10 μM IC_50_ for 48 h: 4.17 ± 1.08 μM IC_50_ for 72 h: 3.02 ± 1.06 μM	Pelorol induced cell growth regression of 501Mel melanoma cells	[30]
5-*epi*-Llimaquinone (**29**)	Prenylquinone	*Dactylospongia elegans*/Sponge	Melanoma cancer	Model: 501Mel Assay: Cell viability assay IC_50_ for 24 h: 7.88 ± 1.08 μM IC_50_ for 48 h: 5.71 ± 1.07 μM IC_50_ for 72 h: 1.71 ± 1.10 μM	5-*epi*-ilimaquinone induced cell growth regression of 501Mel melanoma	[30]

**Table 2:** In-vivo studies of bioactive compounds isolated from marine sponge between 2019 and 2023

Compound name/	Class of compound	Marine source	Type of cancer	Mechanism	References
Siphonodictyal B (**30**)	Meroterpenoid	Aka coralliphaga/Sponge	Colon cancer	Activate the pathway of p38 MAPK and p38 phosphorylation in tumor tissue	[35]
Stellettin B (**31**)	Triterpenoid	Jaspis stellifera/Sponge	Brain cancer	Stellettin B decreases expression of VEGF and inhibits angiogenesis	[36, 37]
Gukulenin A (**32**)	bis-tropolone tetraterpenoid	Phorbas gukhulensis/Sponge	Ovarian cancer	Suppressed ovarian tumor growth via inhibition of MCP-1, RANTES, and VEGF expressions	[38]
Avarol (**33**)	Sesquiterpene hydroquinone	Dysidea avara/Sponge	Ehrlich carcinoma and cervical cancer	Inhibition of tumor growth	[39]

A natural quinazoline derivative named 2-Chloro-6-phenyl-8H-quinazolino[4,3-b]quinazolin-8-one (**1**), was obtained from marine sponge *Hyrtios erectus*. It showed a potentially anticancer impact against human breast cancer as confirmed by MTT assay using MCF-7 as in vitro model. Compound **1** exhibited Half-maximal inhibitory concentration (IC_50_) values of 13.04 ± 1.03 µg/mL and 22.67 ± 1.53 µg/mL for 48 and 24 h, compared with the positive control (cyclophosphamide: IC_50_ values of 8.11 ± 0.84 µg/mL and 15.11 ± 1.16 µg/mL for 48 and 24 h); respectively. The mechanism of action was explained by inducing breast carcinoma cells apoptosis via production of ROS and either extrinsic or intrinsic pathways of apoptosis [23]. In another study, the Indonesian marine sponge of *Spongia* sp. yielded three new bioactive compounds: ceylonamides G–I (**2**–**4**). This study assessed the inhibition of human prostate cancer DU145 cells growth in vitro using 2D monolayer cultures and spheroid of 3D cell culture. Bioactive compound **2** showed a significant effect with IC_50_ 6.9 μM for 2D culture, and Medical Executive Committee (MEC) 10 μM for 3D spheroid cell culture in comparison with taxol with IC_50_ 2.6 nM and MEC 10 nM, respectively. On the other hand, compounds **3** and **4**didn't exhibit activity up to 100 μM. The mode of action for these compounds wasn’t clearly described [24]. Sakai and his team isolated a novel protein from the marine sponge *Spongosorites* sp. named by soritesidine (**5**). Investigation of the cytotoxicity was conducted in vitro through using the cancer cell line HeLa cells and L1210 murine leukemia cells. Soritesidine (**5**) showed a potential IC_50_ value of 0.062 and 12.11 ng/mL, respectively. The mechanism of action for this protein is not investigated yet [25]. Another new cycloheptapeptide named phakefustatin A (**7**), was obtained from *Phakellia fusca* a marine sponge by Wu and co-authors (2020). The cytotoxicity was evaluated against six cancer cell lines of human (HeLa, MCF-7, PC9, NCI-H460, SW480, and HepG2), and (H9c2 and HEK293T) nonmalignant cell lines in vitro. Compound **7** shows a highly significant effect only for HeLa, MCF-7, and NCI-H460 with IC_50_ values of 6.2 ± 0.3, 3.4 ± 1.2, and 7.1 ± 0.6 μM, respectively compared with PC cisplatin has IC_50_ values of 4.4 ± 0.3, 4.8 ± 0.8, and 3.2 ± 1.1 μM, respectively. The mode of action took place through apoptosis and cell growth inhibition via the pathway of Retinoid X receptor alpha (RXRα)-mediated phosphatidylinositol 3-kinase/protein kinase B (PI3K/Akt) signaling [26]. Three bioactive compounds, siphonellamides A and B (8 and 9), two new polyacetylene amides, and siphonellamide E (10), a new fatty amide, were obtained from the Red Sea marine sponge *Siphonochalina siphonella*. These substances were evaluated for cytotoxicity in vitro using MCF-7, HeLa, and A549 cancer cell lines.

. Compounds 8 and 9 demonstrated a strong cytotoxic effect on cancer cell lines. HeLa had IC_50_ values between 9.4 and 17.4 μM, whereas PC 5-Fluorouracil showed IC_50_ values of 28.4 μM.

. According to this study, the acetylene functionality may be responsible for their cytotoxic properties [27]. Surti and co-authors [28] obtained the bioactive compound Ilimaquinone (**17**) from marine sponge *Hippospongia metachromia*. This study evaluated the molecular mechanism of **17** on the anticancer through using colorectal cancer cell line HCT-116.

The results confirmed that compound **17** had a growth-inhibiting effect, with an IC_50_ of 17.89 μM.

Ilimaquinone triggers mitochondria-mediated apoptosis through the decrease in mitochondrial membrane potential and activation caspase-9/-3, DNA damage, and a reduction in Bcl-2 proportion [28]. Two new diterpenoids, kalihioxepane A and B (**18** and **19**), were separated from the marine sponge *Acanthella cavernosa*. The cytotoxic activity was evaluated using five cancer cell lines: K562, ASPC-1, H69AR, H69, and MDA-MB-231. The findings exhibited that exclusively compound **18** displayed a potential effect against K562, ASPC-1, and H69 with IC_50_ values of 6.57, 16.17, and 3.60 μmol/L, compared to positive control doxorubicin with IC_50_ 0.252, 0.023, and 0.980 μmol/L, respectively. While compound **19** showed a potent effect against only one cell line, K562, with IC_50_ 8.73 μmol/L compared with positive control (PC) doxorubicin with IC_50_ 0.252 μmol/L. Both compounds showed significant cytotoxicity, indicating that the isocyano substituent was important [29]. Another study reported two secondary metabolites: pelorol and 5-*epi*-ilimaquinone (**28** and **29**), purified from *Dactylospongia elegans*. Two compounds were evaluated using 501Mel melanoma cells. The findings of the cell viability assay showed that compounds **28** and **29** have a highly significant effect with an IC_50_ value of 3.02 ± 1.06 and 1.71 ± 1.10 μM after 72 h, respectively. In a dependent manner in a concentration and time, the two compounds induced cell growth regression of 501Mel melanoma cells [30]. In combination, 11 bioactive compounds displayed significant effects against different cancer cell lines with IC_50s_ below 20 µM. To obtain potential lead compounds as anticancer treatments, in vivo research is also strongly advised. In this regard, the most potent bioactive compounds require more thorough examination.

#### In vivo studies of bioactive compounds from marine sponge.

Siphonodictyal B (**30**), the biogenetic precursor of liphagal, was purified from the marine sponge *Aka coralliphaga*. In vivo study of colon cancer was conducted on siphonodictyal B (**30**) using the xenograft mouse model (Balb/c nude mice). The intraperitoneal administration (20 mg/kg) of compound **30** every third day showed a potent effect on tumor volume and weight (both significantly smaller) than in the control group. The mode of action was exhibited by the activation of the p38 MAPK pathway and the increase of p38 phosphorylation in tumor tissue [35]. Stellettin B (**31**) is a triterpenoid separated from *Jaspis stellifera* marine sponge. In vivo study of brain cancer was conducted to evaluate stellettin B effect on the inhibition of angiogenesis using a transgenic zebrafish embryo model. The findings showed that the embryos death rate was 0%, 0%, 6%, and 10% in correspondence to stellettin B concentrations of 10, 50 nM, 100, and 250 nM, respectively. While the percentage values of intersegmental vessels (ISVs) were 100% ± 0.5%, 66% ± 9.7%, 68% ± 11.1%, and 25% ± 10.5% of ISVs when **31** was administered at concentrations of 0, 50, 100, and 250 nM, respectively. Stellettin B decreased VEGF expression and caused a decline in VEGF expression as well as angiogenesis inhibition [36, 37]. Anh and his co-authors separated a bioactive compound named gukulenin A (**32**) from marine sponge *Phorbas gukhulensis* and investigated the anticancer activity in vivo using an ovarian cancer xenograft mouse model. Two doses (1 and 3 mg/kg) were applied only once each third day for 15 days and caused tumor growth suppression with 69.30% and 92.43% (inhibition of tumor weight), respectively. The mechanism of action for the compound **32** as an anticancer was explained by suppressing ovarian tumor growth through inhibition of monocyte chemoattractant protein-1 (MCP-1), regulated upon activation, normal T cell expressed and secreted (RANTES), and vascular endothelial growth factor (VEGF) expressions [38]. Avarol (**33**) is a sesquiterpene hydroquinone purified from the marine sponge *Dysidea avara*. In vivo, the study was conducted to investigate avarol effect on cancer using solid Ehrlich carcinoma (EC) and cervical cancer (CC-5) as a model. After three intraperitoneal administrations of (50 mg/kg) avarol exhibited an inhibition rate of 29% and 36% on EC and CC-5 tumor growth, respectively. Similarly, the compound **33** displayed potential antitumor activity via the inhibition of tumor growth [39].

### Secondary metabolites of marine algae against cancer

In accordance with our literature survey, no papers were found to discuss the role of bioactive compounds isolated from marine algae between 2019 and 2023 in treating cancer.

### Secondary metabolites of marine bacteria against cancer.

#### In vitro studies of bioactive compounds from marine bacteria.

**Fig. 5:**
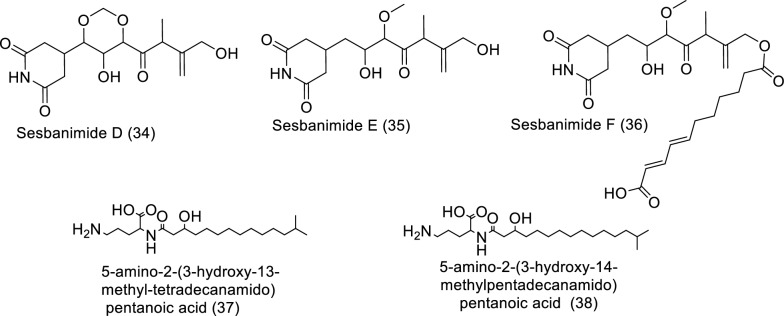
Structures of bioactive compounds isolated from marine bacteria between 2019 and 2023 with in vitro studies

Antagonism is nature's own defense mechanism for surviving and existing. In order to protect themselves from other germs, bacteria develop various secondary metabolites, which are sources of bioactive substances that can be used in human therapeutic procedures. Potential sources of bioactive compounds such as alkaloids, polyketides, polycyclic aromatic hydrocarbons, and nonribosomal peptides (counting for about 70% of those newly found) can be found in the secondary metabolites of marine bacteria, as shown in Table 3 and Fig. 5 [40]. Sesbanimides D-F (**34**–**36**), as well as the known sesbanimides A and C, were separated from two different marine alphaproteobacterial species, namely *Labrenzia aggregata* PHM038 and *Stappia indica* PHM037. The above-mentioned substances significantly reduced the growth of breast, lung, as well as colorectal cancer cell lines [41]. From the *Lacinutrix* species strain, two isobranched lyso-ornithine lipids were found. A 3-hydroxy fatty acid is connected to an ornithine amino acid alpha amino group by an amide bond to form lyso-ornithine lipids, where the fatty acid sequences used were iso-C15:0 named as 5-amino-2-(3-hydroxy-13-methyltetradecanamido) pentanoic acid (**37**) and iso-C16:0 named as 5-amino-2-(3-hydroxy-14-methylpentadecanamido) pentanoic acid (**38**). A2058 human melanoma cells demonstrated cytotoxic activity in response to Lyso-Ornithine lipid **38** [42].

**Table 3:** In vitro studies of bioactive compounds isolated from marine bacteria between 2019 and 2023

Compound Name/	Class of compound	Marine Source	Type of Cancer	Pharmacological effects	Mechanism	References
Sesbanimide D (**34**)	Polyketide	*Labrenzia aggregata* PHM038	Breast cancer, colon cancer, and lung cancer	Model: cell lines of colorectal cancer HT29 (ATCC HTB-38); lung cancer A549 (ATCC CCL-185); and breast adenocarcinoma (ATCC HTB-26) Assay: sulforhodamine B (SRB) GI_50_^*^: 2.0E−08, (Colon HT29), 2.3E−08 (Breast MDA-MB-231), 2.0E−08 (Lung NSCLC A549) Positive control: Doxorubicine	Not reported	[41]
Sesbanimide E (**35**)	Polyketide	*Stappia indica* PHM037	Breast cancer, colon cancer, and lung cancer	Model: lung carcinoma A549 (ATCC CCL-185); colorectal carcinoma HT29 (ATCC HTB-38); and breast cancer MDA-MB-231 (ATCC HTB-26) Assay: sulforhodamine B (SRB) GI_50_: 1.1E−07 (Breast MDA-MB-231, 6.4E−07 (Colon HT29), 6.4E-08 (Lung NSCLC A549) Positive control: Doxorubicine	Not reported	[41]
Sesbanimide F (**36**)	Polyketide	*Stappia indica* PHM037	Lung cancer, colorectal cancer, and breast cancer	Model: lung carcinoma A549; colorectal carcinoma HT29; and breast adenocarcinoma MDA-MB-231 Assay: sulforhodamine B (SRB) GI_50_: 8.6E−09, (Breast MDA-MB-231, 1.1E−08 (Lung NSCLC A549), 1.2E−08 (Colon HT29) Positive control: Doxorubicine	Not reported	[41]
5-amino-2-(3-hydroxy-13-methyltetradecanamido) pentanoic acid(**37**)	phosphorus-free lipids	*Lacinutrix* sp. strain M09B143	Skin cancer	Model: A2058 human melanoma cells (A2058, CRL-1147TM, ATCC) Assay: MTS IC_50_: Not reported Positive control: Not reported	No activity against cells	[42]
5-amino-2-(3-hydroxy-14-methylpentadecanamido) pentanoic acid (**38**)	phosphorus-free lipids	*Lacinutrix* sp. strain M09B143	Skin cancer	Model: A2058 human melanoma cells (CRL-1147TM, ATCC) Assay: MTS IC_50_: Not reported Positive control: Not reported	There was considerable cytotoxic activity against the A2058 cell line, with cell survival rates of 23% at 50 µM, and ~ 0% at 100 and 150 µM	[42]

### Secondary metabolites of marine fungi against cancer

#### In vitro studies of bioactive compounds from marine fungi.

**Fig. 6:**
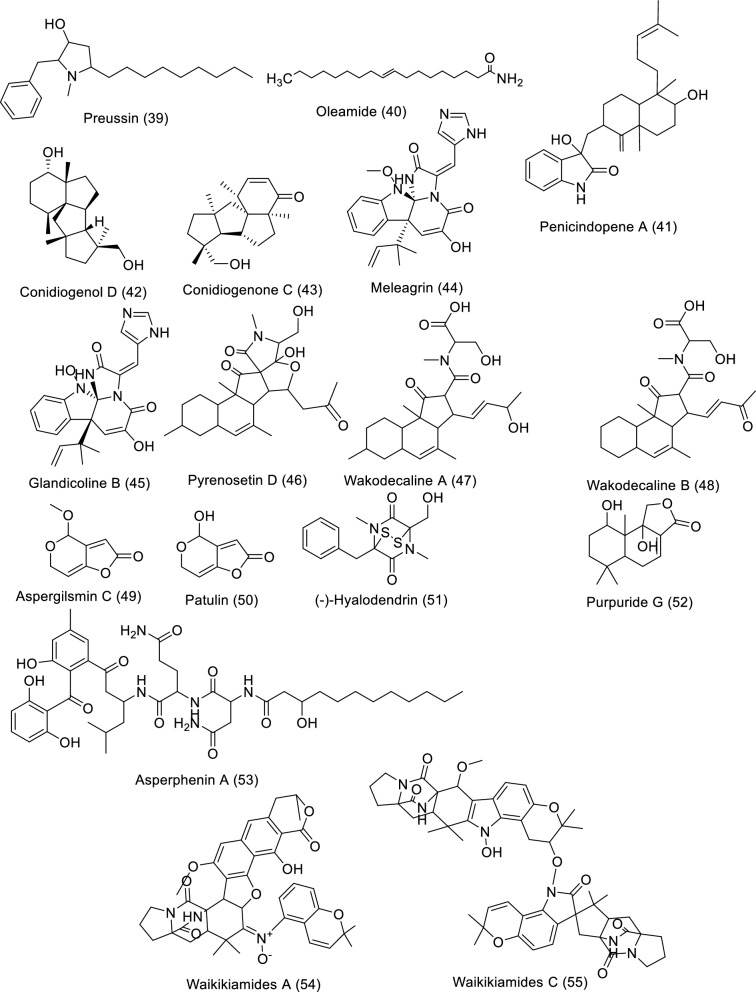

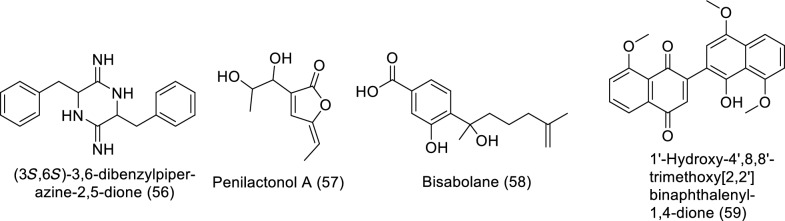
Structures of bioactive compounds isolated from marine fungi between 2019 and 2023 with in vitro studies

One of the primary marine environmental decomposers, marine fungi, has been found to produce distinctive biomolecules and possible enzymes. Preussin (**39**) is a hydroxyl pyrrolidine derivative (Table 4 and Fig. 6) that was found to have anticancer properties in MCF7 and other cancer cell lines after being separated from the fungus *Aspergillus candidus* that is connected to marine sponges (KUFA 0062). The antiproliferative and cytotoxic properties of preussin were examined on breast cancer cell lines (MCF7, SKBR3, and MDA-MB-221) as well as a non-tumor cell line (MCF12A). All examined cell lines were influenced by preussin's effects, as shown by the decline of cell survival and production in 2D and 3D cultures. Within MCF7, MCF12A, and SKBR3, preussin at 25 µM was sufficient to stop cell growth, but not in MDA-MB-231, in which the inhibition only happened at 50 µM [43]. *Penicillium* sp. ArCSPf, a marine sediment-resultant fungus, was separated from the Arabian Sea's eastern continental slope, and its active component of ethyl acetate extract displayed strong anticancer interest (IC_50_ = 22.79 g/mL) regarding MCF-7 breast cancer cells. LC–MS/MS analysis was used to identify the secondary metabolite (Z)-Octadec-9-enamide (oleamide (**40**)) of this fungus' active fraction [44]. *Penicillium* sp. YPCMAC1, a deep-sea fungus, yielded an indole diterpene known as penicindopene A (**41**). According to [45], penicindopene A had reasonable cytotoxicity against A549 and HeLa cell lines, with IC_50_ values of 15.2 and 20.5 µM, respectively. Conidiogenol D (**42**), conidiogenone C (**43**), meleagrin (**44**), and glandicoline B (**45**) were isolated through chemical analysis of an ethyl acetate extract of a deep-sea-derived *Penicillium* sp. All compounds showed a mild inhibitory impact on five esophageal cancer cell lines in the bioassay research, with IC_50_ values varying from 25 to 55 μM [46]. The endophytic bacterium Pyrenochaetopsis FVE-087, which emerged from the Baltic *Fucus vesiculosus*, was shown to include the pentacyclic decalinoylspirotetramic acid derivative pyrenosetin D (**46**), as well as the decalin precursors wakodecalines A (**47**) and B (**48**). These compounds were tested against the non-cancerous keratinocyte (HaCaT) and the human malignant melanoma (A-375) cell lines for their anticancer and toxic possibilities. With IC_50_ values of 77.5 and 39.3 μM, respectively, pyrenosetin D demonstrated toxicity against A-375 and HaCaT cells, whereas wakodecalines A and wakodecalines B were inert [47]. Highly oxygenated polyketides, viz., aspergilsmins A–G, as well as deoxytryptoquivaline, patulin, quinadoline, and tryptoquivaline, were isolated from *Aspergillus* *giganteus* NTU967 extracted from *Ulva lactuca*. Amid these, aspergilsmin C (**49**) and patulin (**50**) exhibited promising anticancer properties against prostate cancer PC-3 cells and human hepatocellular carcinoma SK-Hep-1 cells with IC_50_ values ranging from 2.7 to 7.3 μM [48]. The brown alga *Pelvetia caniculata* served as the source for the marine fungus *Paradendryphiella salina* PC 362H strain, which led to the separation of (-)-hyalodendrin (**51**) as the secondary metabolite responsible for the crude extract's cytotoxic properties. The anticancer effect of (-)-hyalodendrin was evident in cancer cells with spreading characteristics, such as colorectal cancer cells resistive to chemotherapy, and was not just restricted to the MCF7 cell lines. Further research revealed that (-)-hyalodendrin treatment of MCF7-Sh-WISP2 cells changed the phosphorylation level of p53 and changed the expression of HSP60, HSP70, and PRAS40 proteins [49]. The endophytic fungus *Penicillium chrysogenum*, extracted from the marine algae *Chaetomorpha antennina*, possesses anticancer properties that hinder the growth of HeLa cells and alter the apoptotic cell death cycle [50]. With IC_50_ values of 4.5 and 10.9 μM, respectively, purpuride G (**52**) was identified from the marine-sourced fungus *Penicillium minioluteum* ZZ1657, and it significantly suppressed human glioma U257 and U87MG cell lines [51]. A lipopeptidyl benzophenone metabolite called asperphenin A (**53**) was discovered during the marine-derived *Aspergillus* sp. fungus growth process. The substance showed powerful antiproliferative effects on many cancer cells. Asperphenin A stopped the G_2_/M cell cycle and then caused apoptosis in colon cancer cells, preventing them from proliferating. Asperphenin A causes reactive oxygen species in addition to having an impact on the cell cycle. The research also showed that the aryl ketone is crucial in the molecular structure of asperphenin A, which is responsible for its biological activity [52]. The structurally complicated diketopiperazine derivatives waikikiamides A (**54**) and waikikiamides C (**55**) were found in *Aspergillus* sp. FM242. According to [53], the two compounds had antiproliferative activity with IC_50_ values varying from 0.5 to 1.8 μM. The compound (3S,6S)-3,6-dibenzylpiperazine-2,5-dione (**56**) was identified from a culture extract of *Paecilomyces formous* 17D47-2, which is derived from the sea. PANC-1 cells acclimated to conditions of low glucose with an IC_50_ value of 28 µM; however, in normal culture conditions, no effect was seen against PANC-1 cells up to 1000 µM [54]. A pentaketide derivative, penilactonol A (**57**), and sesquiterpenoids of the bisabolane type (**58**), were obtained from the marine alga-related fungus *Penicillium chrysogenum* LD-201810. Human cancer cell lines (BT-549, A549, HeLa, MCF-7, HepG2, and THP-1) were tested for cytotoxicity. Compound **57** had an IC_50_ value of 22.0 μM and was cytotoxic to the HepG2 cell line. With IC_50_ values of 21.2 and 18.2 μM, respectively, 11-dehydrosydonic acid also demonstrated noteworthy activity against A549 and THP-1 cell lines [55]. The marine fungus *Hypoxylon rubiginosum* FS521, which was obtained from a deep-sea deposit sample, was used to make 1′-hydroxy-4′,8,8′-trimethoxy[2,2']binaphthalenyl-1,4-dione (**59**). The compound showed substantial cytotoxic activity with IC_50_ values of 3.2, 1.8, 5.1, and 2.5 μM, respectively, when it was tested for its in vitro cytotoxic activity against the MCF-7, SF-268, A549, and HepG-2 tumor cell lines [56].

**Table 4:** In vitro studies of bioactive compounds isolated from marine fungi between 2019 and 2023

Compound name	Class of compound	Marine source	Type of cancer	Pharmacological effects	Mechanism	References
Preussin (**39**)	Pyrrolidine alkaloid	*Aspergillus candidus* KUFA 0062	Breast cancer	Model: breast cancer cell lines (SKBR3, MCF7, and MDA-MB-231) Assay: MTT, resazurin and lactate dehydrogenase (LDH) and proliferative (5-bromo-2′-deoxyuridine) IC_50_: NR Positive control: NR	Morphological study of preussin-exposed cells revealed cell death	[43]
Oleamide (**40**) amide	Fatty acid	*Penicillium* sp. ArCSPf	Breast cancer	Model: MCF-7 breast cancer cells Assay: MTT IC_50_: 22.8 µg/mL Positive control: NR	–	[44]
Penicindopene A (**41**)	Indole diterpene	*Penicillium* sp. YPCMAC1	Lung cancer	Model: A549 and HeLa cell Assay: MTT IC_50_: 15.2 and 20.5 µM, respectively Positive control: 5-fluorouracil	Not reported	[45]
Conidiogenol D (**42**)	Tetracyclic diterpenes	*Penicillium* sp.	Oesophageal cancer	Model: esophageal cancer cell lines (EC109, EC9706, KYSE30, KYSE70, and KYSE450) Assay: MTT IC_50_:36 to 54 μM Positive control: Cisplatin	Not reported	[46]
Conidiogenone C (**43**)	Diterpenoid	*Penicillium* sp.	Oesophageal cancer	Model: esophageal cancer cell lines (EC109, EC9706, KYSE30, KYSE70, and KYSE450) Assay: MTT IC_50_:27to 42 μM Positive control: Cisplatin	Not reported	[46]
Meleagrin (**44**)	Indole alkaloid	*Penicillium* sp.	Oesophageal cancer	Model: esophageal cancer cell lines (EC109, EC9706, KYSE30, KYSE70, and KYSE450) Assay: MTT IC_50_:25 to 40 μM Positive control: Cisplatin	Not reported	[46]
Glandicoline B (**45**)	Indole alkaloid	*Penicillium* sp.	Oesophageal cancer	Model: esophageal cancer cell lines (EC109, EC9706, KYSE30, KYSE70, and KYSE450) Assay: MTT IC_50_:30 to 55 μM Positive control: Cisplatin	Not reported	[46]
Pyrenosetin D (**46**)	Pentacyclic decalinoyl-spirotetramic acid	*Pyrenochaetopsis* FVE-087	Skin cancer	Model: human malignant melanoma cell line (A-375) Assay: CellTiterBlue Cell Viability Assay IC_50_: 77.5 μM Positive control: Doxorubicine	Not reported	[47]
Wakodecaline A (**47**)	Decaline metabolites	*Pyrenochaetopsis* FVE-087	Skin cancer	Model: human malignant melanoma cell line (A-375) Assay: CellTiterBlue Cell Viability Assay IC_50_: not active Positive control: Doxorubicine	Not reported	[47]
Wakodecaline B (**48**)	Decaline metabolites	*Pyrenochaetopsis* FVE-087	Skin cancer	Model: human malignant melanoma cell line (A-375) Assay: CellTiterBlue Cell Viability Assay IC_50_: not active Positive control: Doxorubicine	Not reported	[47]
Aspergilsmin C (**49**)	Polyketides	*Aspergillus* *giganteus* NTU967	Liver and prostate cancer	Model: human hepatocellular carcinoma SK-Hep-1 cells in addition to prostate cancer PC-3 cells Assay: SRB IC_50_: 2.7 ± 0.2 and 7.3 ± 0.3 μM Positive control: Paclitaxel	Not reported	[48]
Patulin (**50**)/	Polyketide lactone	*Aspergillus* *giganteus* NTU967	Liver and prostate cancer	Model: human hepatocellular carcinoma SK-Hep-1 cells in addition to prostate cancer PC-3 cells Assay: SRB IC_50_: 2.7–7.3 μM Positive control: Paclitaxel	Not reported	[48]
(−)-Hyalodendrin (**51**)	*Epi*-dithiodioxopiperazine	*Paradendryphiella salina* PC 362H	Breast cancer	Model: MCF7 Assay: MTT IC_50_: 0.1 μM Positive control: hydroxy tamoxifen	MCF7-Sh-WISP2 cells with (-)-hyalodendrin induced alterations in the phosphorylation state of p53 and changed the expression of HSP70, HSP60 and PRAS40 proteins	[49]
Purpuride G (**52**)	Drimane sesquiterpenoids	*Penicillium minioluteum* ZZ1657	Brain cancer	Model: human glioma U257 U87MG cell lines, Assay: MTT IC_50_: 4.5 and 10.9 μM Positive control: -	Not reported	[51]
Asperphenin A (**53**)	Lipopeptidyl benzophenone metabolite	*Penicillium* sp.	Colon cancer	Model: RKO colon cancer cells Assay: sulforhodamine B (SRB) IC_50_: 0.8 μM Positive control: Etoposide	Asperphenin A stopped the growth of colon cancer cells via G_2_/M cell cycle arrest and apoptosis. In addition to its impact on cell cycle, asperphenin A triggered reactive oxygen species	[52]
Waikikiamide A (**54**)	Diketopiperazine Dimer	*Aspergillus* sp. FM242	Diverse	Model: HT1080, Pc3, Jurkat and A2780S Assay: MTT IC_50_: 0.6 to 1.8 μM Positive control: taxol	Not reported	[53]
Waikikiamide C (**55**)	Diketopiperazine Dimer	*Aspergillus* sp. FM242	Diverse	Model: HT1080, Pc3, Jurkat and A2780S Assay: MTT IC_50_: 1.1 to 1.8 μM Positive control: taxol	Not reported	[53]
(3*S*,6*S*)-3,6-dibenzylpiperazine-2,5-dione (**56**)	*Dibenzylpiperazines*	*Paecilomyces formous* 17D47-2	Pancreatic Cancer	Model: human pancreatic carcinoma PANC-1 cells Assay: MTT IC_50_: 28 µM Positive control: taxol	Not reported	[54]
Penilactonol A** (57)**	Pentaketide	*Penicillium chrysogenum* LD-201810	Diverse	Model: human carcinoma cell lines (BT-549, A549, HepG2, HeLa, THP-1, and MCF-7) Assay: MTT IC_50_: 22.0 μM against HepG2 Positive control: taxol	Not reported	[55]
Bisabolane** (58)**	Sesquiterpenes	*Penicillium chrysogenum* LD-201810	Diverse	Model: human lung adenocarcinoma epithelial cell line A549, human cervix carcinoma cell line HeLa, human breast cancer cell line BT-549, human liver carcinoma cell line HepG2, and human monocytic cell line THP-1, and human breast adenocarcinoma cell line MCF-7 Assay: MTT IC_50_: 21.2 and 18.2 μM against A549 and THP-1 cell lines Positive control: Epirubicin	Not reported	[55]
1′-Hydroxy-4′,8,8′-trimethoxy[2,2′]binaphthalenyl-1,4-dione (**59**)	Quinones	*Hypoxylon rubiginosum* FS521	Diverse	Model: SF-268, FS521, MCF-7, A549, and HepG-2 tumor cell lines Assay: SRB IC_50_: 1.8, 2.5, 3.2, and 5.1 mM against SF-268, HepG-2, MCF-7, and A549 cell lines, respectively Positive control: cisplatin	Not reported	[56]

#### In vivo studies of bioactive compounds from marine fungi.

**Fig. 7:**
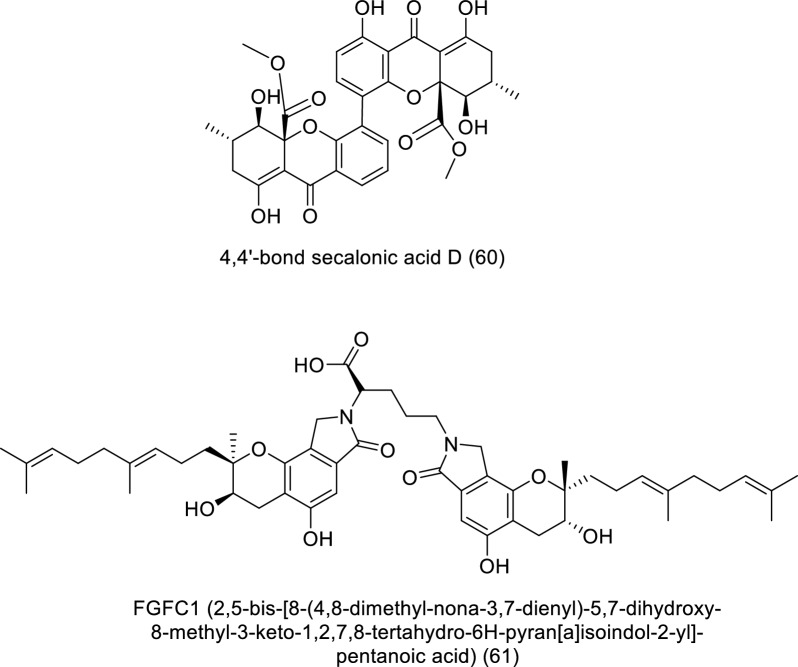
Structures of bioactive compounds isolated from marine fungi between 2019 and 2023 with in vivo studies

The effect of FGFC1 (**61**) isolated from marine fungi *Stachybotrys longispora* FG216 on PC9 tumor transplant growth in BALB/c athymic nude mice was discussed as shown in Table 5 and Fig. 7 [57]. The study demonstrated that FGFC1 could inhibit PC9 cell development via controlling NF-κB signaling pathways, with no detectable effects on the mice's overall body weight.

**Table 5:** In vivo studies of bioactive compounds isolated from marine fungi between 2019 and 2023

Compound Name	Class of compound	Marine Source	Type of Cancer	Mechanism	References
4,4′-bond secalonic acid D (**60**)	Xanthene	*Penicillium oxalicum*	hepatocellular carcinoma	It regulated Bax expression, which is a biomarker of tumor growth	[58]
FGFC1 (2,5-bis-[8-(4,8-dimethyl-nona-3,7-dienyl)-5,7-dihydroxy-8-methyl-3-keto-1,2,7,8-tertahydro-6H-pyran[a]isoindol-2-yl]-pentanoic acid) (**61**)/	Isoindolone alkaloid	*Stachybotrys longispora* FG216 (CCTCCM 2012272)	Lung cancer	Without significantly affecting the mice's overall body weight, FGFC1 therapy significantly inhibits PC9 cell development through controlling NF-κB signalling pathways	[57]

### Secondary metabolites of marine soft corals against cancer

**Fig. 8:**
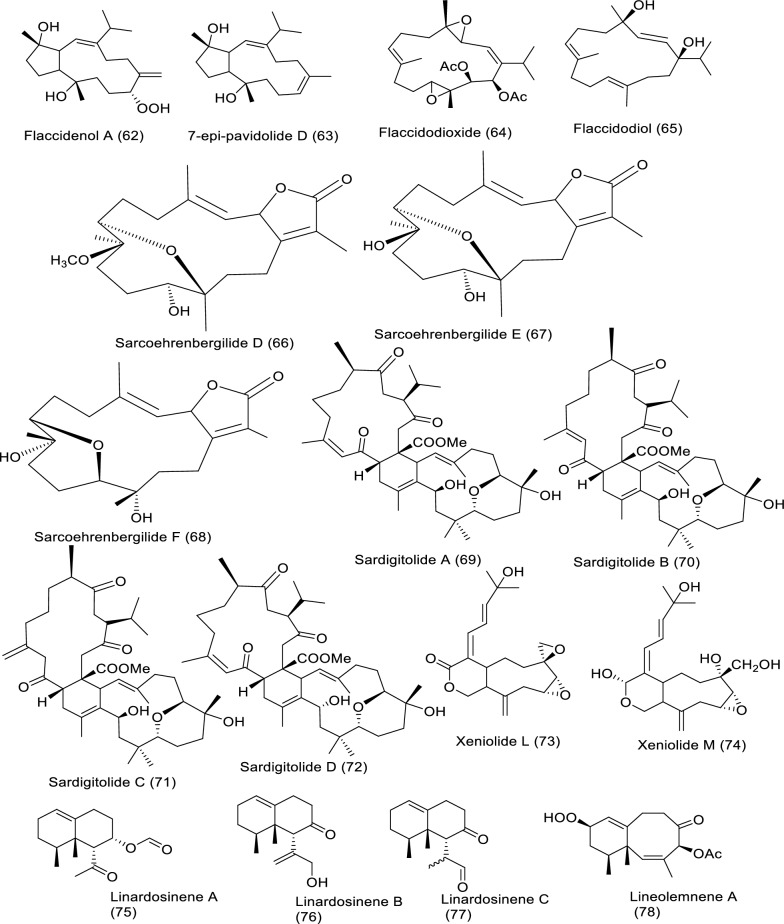

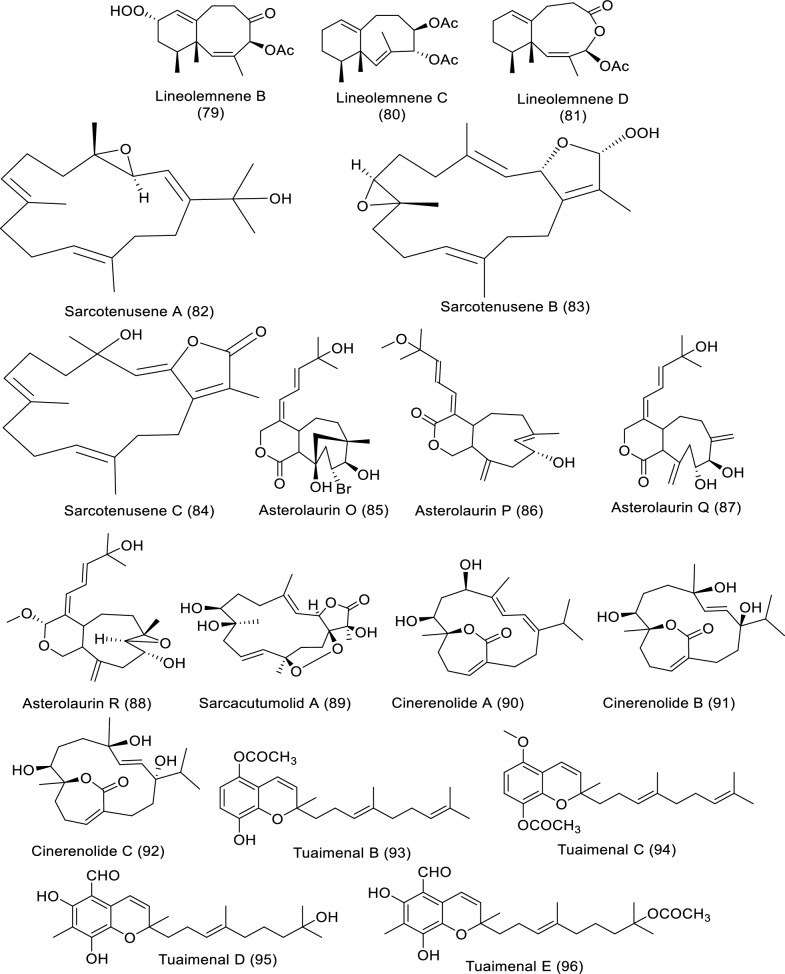

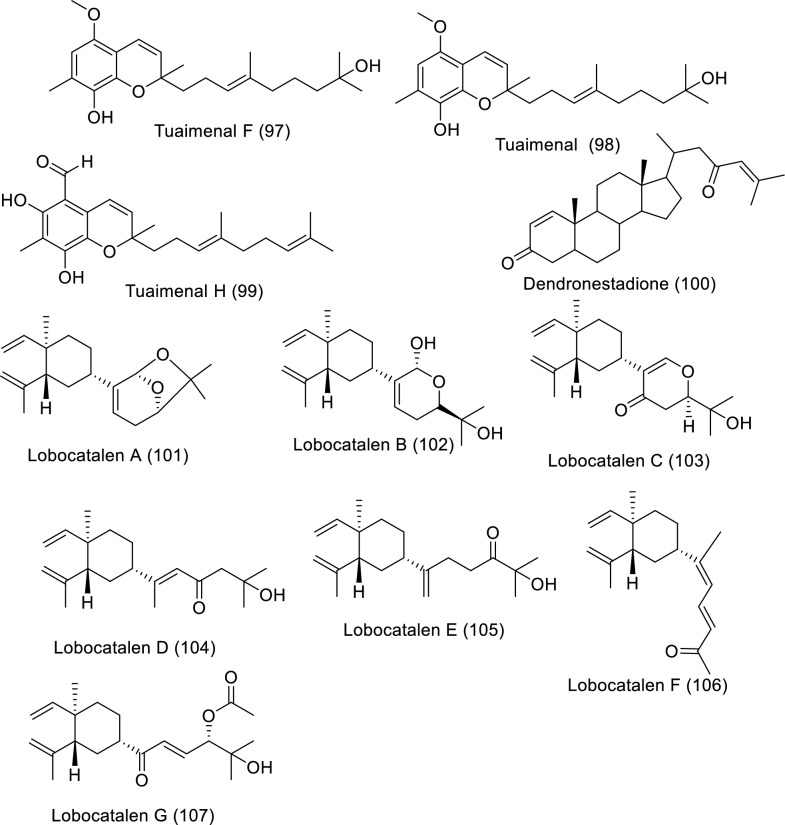
Structures of bioactive compounds isolated from marine soft corals between 2019 and 2023 with in vitro studies

Soft corals are considered one of the richest sources of bioactive products, especially diterpenes, triterpenes, and steroids. Soft coral-derived metabolites have effective anticancer bioactivities against several cancer types [17]. Bioactive molecules extracted from marine soft corals and their impact against diverse cancer cell lines in in vitro studies are illustrated in Table 6 and Fig. 8.

**Table 6:** In vitro studies of bioactive compounds isolated from marine soft corals between 2019 and2023

Compound name	Class of compound	Marine source	Type of cancer	Pharmacological effects	Mechanism	References
Flaccidenol A (**62**)	Cembrane-derived diterpenoid	*Klyxum flaccidum*	Human lung, colorectal carcinoma, and mouse lymphocytic leukemia	Model: A549, DLD-1, P388D1 Assay: MTT IC_50_: 9.7 ± 1.2 μg/mL to A549, 6.0 ± 0.4 μg/mL to DLD-1, 7.2 ± 1.8 μg/mL to P388D1 PC: doxorubicin	Not reported	[60]
7-*epi*-Pavidolide D (**63**)	Cembrane-derived diterpenoid	*Klyxum flaccidum*	Human lung, colorectal carcinoma, and mouse lymphocytic leukemia	Model: A549, DLD-1, P388D1 Assay: MTT IC_50_: 28.6 ± 3.8 μg/mL to A549, 31.6 ± 3.7 μg/mL to DLD-1, 30.4 ± 4.8 μg/mL to P388D1 PC: doxorubicin	Not reported	[60]
Flaccidodioxide (**64**)	Cembrane-derived diterpenoid	*Klyxum flaccidum*	Human lung, colorectal carcinoma, and mouse lymphocytic leukemia	Model: A549, DLD-1, P388D1 Assay: MTT IC_50_: 19.6 ± 8.3 μg/mL to P388D1 PC: doxorubicin	Not reported	[60]
Flaccidodiol (**65**)/	Cembrane-derived diterpenoid	*Klyxum flaccidum*	Human lung, colorectal carcinoma, and mouse lymphocytic leukemia	Model: A549, DLD-1, P388D1 Assay: MTT IC_50_: not reported PC: doxorubicin	Not reported	[60]
Sarcoehrenbergilide D (**66**)	Cembrene diterpenoid	*Sarcophyton ehrenbergi*	Human lung carcinoma, colon and hepatic cancer	Model: A549, Caco-2, HepG2 Assay: MTT IC_50_: 23.3 mM to A549 PC: not reported	Not reported	[61]
Sarcoehrenbergilide E (**67**)	Cembrene diterpenoid	*Sarcophyton ehrenbergi*	Human lung carcinoma, colon and hepatic cancer	Model: A549, Caco-2, HepG2 Assay: MTT IC_25_: 27.3 mM to A549 22.6 mM to HepG2 PC: not reported	Not reported	[61]
Sarcoehrenbergilide F (**68**)	Cembrene diterpenoid	*Sarcophyton ehrenbergi*	Human lung carcinoma, colon and hepatic cancer	Model: A549, Caco-2, HepG2 Assay: MTT IC_25_: 25.4 mM to A549 31.8 mM to HepG2 PC: not reported	Not reported	[61]
Sardigitolide A (**69**)	Biscembranoid	*Sarcophyton digitatum*	Breast cancer, breast adenocarcinoma, hepatic and cervical cancer	Model: MCF-7, MDA-MB-231, HepG2, and HeLa Assay: MTT IC_50_: not reported PC: doxorubicin	Not reported	[62]
Sardigitolide B (**70**)	Biscembranoid	*Sarcophyton digitatum*	Breast cancer, breast adenocarcinoma, hepatic and cervical cancer	Model: MCF-7, MDA-MB-231, HepG2, and HeLa Assay: MTT IC_50_: 9.6 ± 3.0 µg/mL to MCF-7 14.8 ± 4.0 µg/mL to MDA-MB-231 PC: doxorubicin	Not reported	[62]
Sardigitolide C (**71**)	Biscembranoid	*Sarcophyton digitatum*	Breast cancer, breast adenocarcinoma, hepatic and cervical cancer	Model: MCF-7, MDA-MB-231, HepG2, and HeLa Assay: MTT IC_50_: not reported PC: doxorubicin	Not reported	[62]
Sardigitolide D (**72**)	Biscembranoid	*Sarcophyton digitatum*	Breast cancer, breast adenocarcinoma, hepatic and cervical cancer	Model: MCF-7, MDA-MB-231, HepG2, and HeLa Assay: MTT IC_50_: not reported PC: doxorubicin	Not reported	[62]
Xeniolide L (**73)**	Xeniolide diterpene	*Xenia umbellate*	Human liver, prostate and colon cancer	Model: HepG2, PC‑3, and HT‑29 Assay: SRB IC_50_: 36.8 ± 1.1 to HepG2 24.9 ± 1.3 to PC-3 13.9 ± 2.5 μg/mL to HT-29 PC: Doxorubicin	Induction of apoptosis	[63]
Xeniolide M (**74**)/	Xeniolide diterpene	*Xenia umbellate*	Human liver, prostate and colon cancer	Model: HepG2, PC‑3, and HT‑29 Assay: SRB IC_50_: 14.7 ± 0.4 to HepG2, 10.9 ± 0.5 to PC‑3, 4.7 ± 0.5 μg/mL to HT-29 PC: Doxorubicin	Induction of apoptosis	[63]
Linardosinene A (**75**)	Nardosinane-type sesquiterpenoid	*Litophyton nigrum*	Human lung epithelial, hepatocellular, colon, pancreatic cancer, and lung cancer	Model: THP-1, SNU-398, HT-29, Capan-1 and A549 Assay: MTT IC_50_: not reported PC: Vincristine	Not reported	[64]
Linardosinene B (**76**)	Nardosinane-type sesquiterpenoid	*Litophyton nigrum*	Human lung epithelial, hepatocellular, colon, pancreatic cancer, and lung cancer	Model: THP-1, SNU-398, HT-29, Capan-1 and A549 Assay: MTT IC_50_: 59.5 μM to THP-1 PC: Vincristine	Not reported	[64]
Linardosinene C (**77**)	Nardosinane-type sesquiterpenoid	*Litophyton nigrum*	Human lung epithelial, hepatocellular, colon, pancreatic cancer, and lung cancer	Model: THP-1, SNU-398, HT-29, Capan-1 and A549 Assay: MTT IC_50_: 24.3 μM to SNU-398 44.7 μM to HT-29 > 50 to Capan-1 and A549 PC: Vincristine	Not reported	[64]
Lineolemnene A (**78**)	Neolemnane-type sesquiterpenoid	*Litophyton nigrum*	Human lung epithelial, hepatocellular, colon, pancreatic cancer, and lung cancer	Model: THP-1, SNU-398, HT-29, Capan-1 and A549 Assay: MTT IC_50_: 44.4 μM to SNU-398 > 50 to Capan-1, A549 and HT-29 PC: Vincristine	Not reported	[64]
Lineolemnene B (**79**)	Neolemnane-type sesquiterpenoid	*Litophyton nigrum*	Human lung epithelial, hepatocellular, colon, pancreatic cancer, and lung cancer	Model: THP-1, SNU-398, HT-29, Capan-1 and A549 Assay: MTT IC_50_: 27.6 μM to SNU-398 > 50 to Capan-1, A549 and HT-29 PC: Vincristine	Not reported	[64]
Lineolemnene C (**80**)	Neolemnane-type sesquiterpenoid	*Litophyton nigrum*	Human lung epithelial, hepatocellular, colon, pancreatic cancer, and lung cancer	Model: THP-1, SNU-398, HT-29, Capan-1 and A549 Assay: MTT IC_50_: not reported PC: Vincristine	Not reported	[64]
Lineolemnene D (**81**)	Neolemnane-type sesquiterpenoid	*Litophyton nigrum*	Human lung epithelial, hepatocellular, colon, pancreatic cancer, and lung cancer	Model: THP-1, SNU-398, HT-29, Capan-1 and A549 Assay: MTT IC_50_: not reported PC: Vincristine	Not reported	[64]
Sarcotenusene A (**82**)	Cembranoid diterpenoid	*Sarcophyton tenuispiculatum*	Breast cancer, human breast adenocarcinoma, hepatocellular carcinoma and cervical cancer	Model: MCF-7, MDA-MB-231, HepG2, and HeLa Assay: MTT IC_50_: 34.3 ± 3.7 µm to MCF-7 PC: doxorubicin	Not reported	[65]
Sarcotenusene B (**83**)	Cembranoid diterpenoid	*Sarcophyton tenuispiculatum*	Breast cancer, human breast adenocarcinoma, hepatocellular carcinoma and cervical cancer	Model: MCF-7, MDA-MB-231, HepG2, and HeLa Assay: MTT IC_50_: not reported PC: doxorubicin	Not reported	[65]
Sarcotenusene C (**84**)	Cembranoid diterpenoid	*Sarcophyton tenuispiculatum*	Breast cancer, human breast adenocarcinoma, hepatocellular carcinoma and cervical cancer	Model: MCF-7, MDA-MB-231, HepG2, and HeLa Assay: MTT IC_50_: not reported PC: doxorubicin	Not reported	[65]
Asterolaurin O (**85**)	Xenicane diterpenoid	*Asterospicularia laurae*	Oral, breast, and ovarian cancer	Model: MCF-7, Ca9-22, SK-OV-3 Assay: MTS IC_50_: 14.7 ± 0.2 µM to MCF-7 > 100 µM to other cells PC: Cisplatin	Not reported	[66]
Asterolaurin P (**86**)	Xenicane diterpenoid	*Asterospicularia laurae*	Oral, breast, and ovarian cancer	Model: MCF-7, Ca9-22, SK-OV-3 Assay: MTS IC_50_: 25.1 ± 4.1 µM for to MCF-7 > 100 µM to other cells PC: Cisplatin	Not reported	[66]
Asterolaurin Q (**87**)	Xenicane diterpenoid	*Asterospicularia laurae*	Oral, breast, and ovarian cancer	Model: MCF-7, Ca9-22, SK-OV-3 Assay: MTS IC_50_: > 100 µM PC: Cisplatin	Not reported	[66]
Asterolaurins R (**88**)	Xenicane diterpenoid	*Asterospicularia laurae*	Oral, breast, and ovarian cancer	Model: MCF-7, Ca9-22, SK-OV-3 Assay: MTS IC_50_: > 100 µM PC: Cisplatin	Not reported	[66]
Sarcacutumolid A (**89**)	Cembranolide	*Sarcophyton acutum*	Hepatocellular, cervical cancer, breast adenocarcinoma and colorectal cancer	Model: HepG2, HeLa, MCF-7 and Colo-205 Assay: SRB IC_50_: 35.5 µM PC: doxorubicin HCl (Dox)	Not reported	[67]
Cinerenolide A (**90**)	Cembranolide	*Sarcophyton cinereum*	Human colorectal adenocarcinoma, intrahepatic cholangiocarcinoma, human skin fibroblast and Mouse lymphoma	Model: P388, DLD-1, HuCCT-1, CCD966SK Assay: Alamar Blue (resazurin) IC_50_: not reported PC: doxorubicin	Not reported	[71]
Cinerenolide B (**91**)	Cembranolide	*Sarcophyton cinereum*	Human colorectal adenocarcinoma, intrahepatic cholangiocarcinoma, human skin fibroblast and Mouse lymphoma	Model: P388, DLD-1, HuCCT-1, CCD966SK Assay: Alamar Blue (resazurin) IC_50_: > 30 µM PC: doxorubicin	Not reported	[71]
Cinerenolide C (**92**)	Cembranolide	*Sarcophyton cinereum*	Human colorectal adenocarcinoma, intrahepatic cholangiocarcinoma, human skin fibroblast and Mouse lymphoma	Model: P388, DLD-1, HuCCT-1, CCD966SK Assay: Alamar Blue (resazurin) IC_50_: > 30 µM PC: doxorubicin	Not reported	[71]
Tuaimenal B (**93**)	Merosesquiterpene	*Duva florida*	Cervical cancer	Model: CaSki and C33A Assay: ELISA IC_50_: 25 μM to CaSki 14 μM to C33A PC: Etoposide	Not reported	[68]
Tuaimenal C (**94**)	Merosesquiterpene	*Duva florida*	Cervical cancer	Model: CaSki and C33A Assay: ELISA IC_50_: not reported PC: Etoposide	Not reported	[68]
Tuaimenal D (**95**)	Merosesquiterpene	*Duva florida*	Cervical cancer	Model: CaSki and C33A Assay: ELISA IC_50_: not reported PC: etoposide	Not reported	[68]
Tuaimenal E (**96**)	Merosesquiterpene	*Duva florida*	Cervical cancer	Model: CaSki and C33A Assay: ELISA IC_50_: not reported PC: etoposide	Not reported	[68]
Tuaimenal F (**97**)	Merosesquiterpene	*Duva florida*	Cervical cancer	Model: CaSki and C33A Assay: ELISA IC_50_: 41 μM to CaSki 38 μM to C33A PC: etoposide	Not reported	[68]
Tuaimenal G (**98**)	Merosesquiterpene	*Duva florida*	Cervical cancer	Model: CaSki and C33A Assay: ELISA IC_50_: 20 μM to CaSki 0.04 μM to C33A PC: etoposide	Not reported	[68]
Tuaimenal H (**99**)	Merosesquiterpene	*Duva florida*	Cervical cancer	Model: CaSki and C33A Assay: ELISA IC_50_: 23 μM to CaSki 14 μM to C33A PC: etoposide	Not reported	[68]
Dendronestadione (**100**)	Ketosteroids	*Dendronephthya* sp.	Human hepatocellular carcinoma, colorectal carcinoma and prostate carcinoma	Model: HepG2, HT-29, PC Assay: MTT IC_50_: 19.1 ± 1.8 μM to HepG2, 32.4 ± 2.8 μM to HT-29, 7.8 ± 0.8 μM to PC-3 PC: doxorubicin	Not reported	[69]
Lobocatalen A (**101**)	Lobane diterpenoid	*Lobophytum catalai*	Human leukemia, pancreatic cancer	Model: K562, ASPC-1, MDA-MB-231 Assay: MTT and SRB IC_50_: > 30 µM PC: doxorubicin	Not reported	[70]
Lobocatalen B (**102**)	Lobane diterpenoid	*L. catalai*	Human leukemia, pancreatic cancer	Model: K562, ASPC-1, MDA-MB-231 Assay: MTT and SRB IC_50_: > 30 µM PC: doxorubicin	Not reported	[70]
Lobocatalen C (**103**)	Lobane diterpenoid	*L. catalai*	Human leukemia, pancreatic cancer	Model: K562, ASPC-1, MDA-MB-231 Assay: MTT and SRB IC_50_: > 30 µM PC: doxorubicin	Not reported	[70]
Lobocatalen D (**104**)	Lobane diterpenoid	*L. catalai*	Human leukemia, pancreatic cancer	Model: K562, ASPC-1, MDA-MB-231 Assay: MTT and SRB IC_50_: > 30 µM PC: doxorubicin	Not reported	[70]
Lobocatalen E (**105**)	Lobane diterpenoid	*L. catalai*	Human leukemia, pancreatic cancer	Model: K562, ASPC-1, MDA-MB-231 Assay: MTT and SRB IC_50_: > 30 µM PC: doxorubicin	Not reported	[70]
Lobocatalen F (**106**)	Lobane diterpenoid	*L. catalai*	Human leukemia, pancreatic cancer	Model: K562, ASPC-1, MDA-MB-231 Assay: MTT and SRB IC_50_: > 30 µM PC: doxorubicin	Not reported	[70]
Lobocatalen G (**107**)	Lobane diterpenoid	*L. catalai*	Human leukemia, pancreatic cancer	Model: K562, ASPC-1, MDA-MB-231 Assay: MTT and SRB IC_50_: 27.9 µM to K562 > 30 µM to other cells PC: doxorubicin	Not reported	[70]

*Sarcophyton* sp. afforded different bioactive metabolites with great biological activities. Major bioactive metabolites of soft corals are terpenes, molecules made up of isoprene building blocks, which undergo modification by re-arrangement or oxidation to form terpenoids. Because of their vast diversity, terpenoids are used for the treatment of many diseases, including cancer. In vitro studies revealed that marine-extracted secondary metabolites exerted the anticancer properties via suppression of protein synthesis and cell cycle inhibition, in addition to induction of programmed cell death [59]. Soft coral *Klyxum flaccidum* extracted cembrane diterpenoids such as flaccidenol A (**62**) and 7-*epi*-pavidolide D (**63**), flaccidodioxide (**64**), and flaccidodiol (**65**) were examined for cytotoxicity to human colorectal adenocarcinoma (DLD-1), lung adenocarcinoma (A549), and mouse lymphocytic leukemia (P388D1). Flaccidenol A as well as 7-*epi*-pavidolide D exhibited anti-proliferative activities against the screened cancer cells. The cytotoxic effects could be related to structure –function dependence; in other words, the presence of hydroperoxyl (as in flaccidenol A) enhanced the cytotoxic potency of the diterpenoid molecules [60]. Sarcoehrenbergilides D–F (**66**–**68**), cembrene–derived diterpenoids, extracted from *Sarcophyton ehrenbergi* were tested against human cancer cell lines like liver (HepG2), colon (Caco-2), and lung (A549). Bioassays revealed that A549 cell viability was inhibited by Sarcoehrenbergilides D–F, whereas HepG2 growth was slightly affected by Sarcoehrenbergilides E–F [61]. Cytotoxicity of Sarcophyton digitatum–isolated sardigitolides A–D (**69**–**72**) were evaluated against MCF-7, MDA-MB-231, HepG2, and HeLa cells. Sardigitolide B displayed anti-proliferative effects on breast cancer cell lines [62]. Xeniolides L (**73**) and M (**74**) were isolated from *Xenia umbellate* and evaluated for viability suppression of HepG2, PC‑3, and HT‑29, exhibiting potent anti-proliferative effects. Features of apoptosis were observed in both HepG2 and PC‑3 after treatment with xeniolide L, whereas exposure to xeniolide M produced apoptotic effects in HepG2 cells [63]. *Litophyton nigrum* isolated–linardosinenes A–C (**75**–**77**) and lineolemnenes A–D (**78**–**81**) were tested for their anti-proliferation against human lung epithelial carcinoma, THP-1, hepatocellular carcinoma, SNU-398, colon carcinoma, HT-29, pancreatic cancer, Capan-1 and lung cancer, A549, tumor cells. The results indicated that linardosinene B inhibited proliferation of THP-1, while linardosinene C and lineolemnene B were cytotoxic to SNU-398, as well as linardosinene C was cytotoxic toward HT-29 cell lines [64]. New cembranoids, sarcotenusenes A–C (**82**–**84**), extracted from *Sarcophyton tenuispiculatum*, were evaluated against MCF-7, MDA-MB-231, HepG2, and HeLa. The results demonstrated sarcotenusene A was a cytotoxic breast cancer cell line [65]. Newly discovered diterpenoids, asterolaurins O–R (**85**–**88**), were isolated from *Asterospicularia laurae* and examined for anti-proliferative potentials in MCF-7 (breast), Ca9-22 (oral), and SK-OV-3 (ovarian) cancer cells. Asterolaurins O–P suppressed proliferation of MCF-7 cell and strong activities were observed by asterolaurin O [66]. Sarcacutumolid A (**89**) was tested for anti-proliferative activity against human HepG-2 (liver), HeLa (cervix), and MCF-7 (breast) cell lines. Sarcacutumolid A exhibited anti-proliferative impact against colorectal cancer (Colo-205) [67]. Tuaimenals B–H (**93**–**99**), derived from *Duva florida* showed growth arrest to cervical cancer CaSki and C33A cell lines, Tuaimenals B, F, and G displayed forceful toxicity against the C33A cells [68]. Dendronestadione (**100**) extracted from soft coral *Dendronephthya* sp. showed significant cellular toxicity to a collection of human cancer cells made up of HepG2 (hepatocellular carcinoma), HT-29 (colorectal carcinoma), as well as PC (prostate carcinoma). Dendronestadione revealed a high effect on cancer cell lines [69]. *Lobophytum catalai*–isolated Lobocatalens A–G (**101**–**107**) cytotoxicity was evaluated to the human leukemia (K562), pancreatic (ASPC-1), and breast (MDA-MB-231) cancer cell lines. Cell viability assay showed lobocatalens G to be cytotoxic toward K562 human cancer cell line [70]*.*

### Secondary metabolites of marine actinomycetes against cancer

**Fig. 9:**
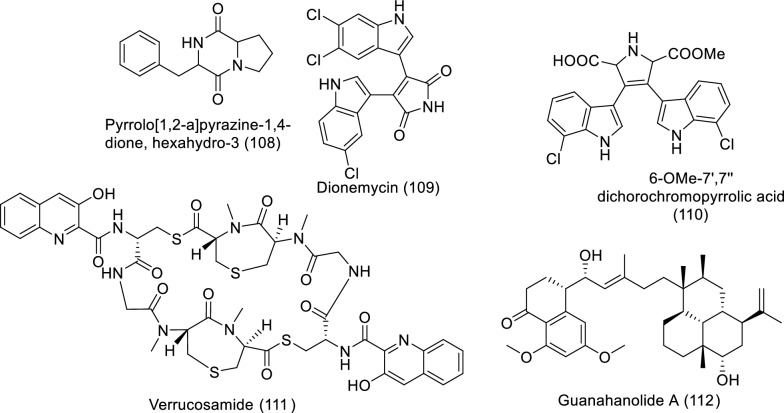
Structures of bioactive compounds isolated from marine actinomycetes between 2019 and 2023 with in vitro studies

Actinomycetes, Gram-positive filamentous bacteria, are capable of producing various bioactive secondary metabolites, including anti-proliferative, cytotoxic, or antitumor molecules [72]. The secondary metabolites of marine actinomycetes that were identified, isolated, or classified as anticancer on in vitro models between 2019 and 2023 are displayed in Table 7 and Fig. 9.

**Table 7:** In vitro studies of bioactive compounds isolated from marine actinomycin between 2019 and 2023

Compound name	Class of compound	Marine source	Type of cancer	Pharmacological effects	Mechanism	References
Pyrrolo[1,2-a]pyrazine-1,4-dione, hexahydro-3 (**108**)	Pyrrolopyrazines	Akiyoshiensis GRG 6 (KY457710)	Breast cancer	Model: MCF-7 Assay: MTT IC_50_: 150 mg/mL PC: MCF-7 (untreated)	Induction of apoptosis	[74]
Dionemycin (**109**)	Chlorinated bis-indole alkaloid	*Streptomyces* sp. SCSIO 11,791	Breast cancer, lung cancer, colon cancer, liver cancer	Model: MDAMB-435, MDA-MB-231, NCI-H460, HCT-116, HepG2 and MCF10A Assay: MTT IC_50_: 3.9 to MDAMB-435 11.2 to MDA-MB-231, 3.6 to NCI-H460, 4.3 HCT-116, 8.2 to HepG2, 3.1 µm to MCF10A PC: EPI (epirubicin)	Not reported	[78]
6-OMe-7′,7′′-dichorochromopyrrolic acid (**110**)	Chlorinated bis-indole alkaloid	*Streptomyces* sp. SCSIO 11,791	Breast cancer, lung cancer, colon cancer, liver cancer	Model: MDAMB-435, MDA-MB-231, NCI-H460, HCT-116, HepG2 and MCF10A Assay: MTT IC_50_: 19.4 to MDAMB-435, > 25.0 to MDA-MB-231, > 25.0 to NCI-H460, 13.1 to HCT-116, 18.5 to HepG2, 2.9 µm to MCF10A PC: EPI (epirubicin)	Not reported	[78]
Verrucosamide (**111**)	Thiodepsipeptide	Marine-derived actinomycete, a *Verrucosispora* sp. strain CNX-026,	Breast carcinoma and colon adenocarcinoma	Model: NCI 60 cell lines Assay: not reported IC_50_: 1.2 µM & 1.4 µM for MDA-MB-468 & COLO 205 respectively PC: not reported	Not reported	[79]
Guanahanolide A (**112**)	Sesterterpene meroterpenoid	*Streptomyces* sp. RKBHB7	Breast cancer, colon cancer	Model: MCF-7, NCI-60, HCT-116, HTB-26 and Vero cell Assay: IC_50_ for MCF-7: 7.8 μM IC_50_ for NCI-60: Panel at 10.0 μM IC_50_ for HCT-116: 11.9 μM IC_50_ for HTB-26: 10.1 μM IC_50_ for Vero cell: 23.7 μM PC: NR	Not reported	[80]

The bulk of actinomycetes are hosted by marine sponges, specially Streptomyces being the most abundant genus [73] which has the potential to produce various novel bioactive compounds [73, 74]. Streptomyces have several verified antitumor molecules like bleomycin, dactinomycin, mitomycin, and doxorubicin [75, 76]. The anticancer activity of marine actinomycetes is attributed mainly to cytotoxic alkaloids [77]. The bioactive anticancer molecules act selectively toward malignant cells, leaving normal cells with minimal cytotoxic impact [73]. *Streptomyces* sp. VITSDK1 and LCJ85 showed potent anti-proliferative properties, and they effectively inhibit angiogenesis. Another study revealed that pyrrolo[1,2-a]pyrazine-1,4-dione, hexahydro-3 (**108**) extracted from marine actinomycetes *Streptomyces akiyoshiensis* GRG 6 (KY457710) was a cancer inhibitor to breast cancer cells MCF-7 (Nadar Rajivgandhi et al., 2020). Moreover, *Streptomyces* sp. SCSIO 11,791–isolated secondary metabolites have potent anticancer effects. Dionemycin (**109**) and 6-OMe-7′,7′′-dichorochromopyrrolic acid (**110**), chlorine and indole-containing alkaloids, were toxic to HepG2, MD1-MB-435, MCF10A, and HCT-116 cell lines. Furthermore, the bioactivity of dionemycin manifested cytotoxic criteria against MDA-MB-231 and NCI-H460 cell lines [78]. Nair and his colleges (2020) isolated a new cytotoxic thiodepsipeptide, verrucosamide (**111**), extracted from marine *Verrucosispora* sp. (CNX-026) and evaluated its cytotoxic effects on the NCI 60 cell line. Colon adenocarcinoma (COLO 205) and breast carcinoma (MDA-MB-468) cells showed significant sensitivity to the doses among the other cancer cell lines in the panel. The cytotoxicity was assigned to verrucosamide and related metabolite thiocoraline, which is also isolated from various strains of marine *Verrucosispora* sp. [79]. Recently, researchers extracted a meroterpenoid, guanahanolide A (**112**), from *Streptomyce*s sp. RKBHB7 and elucidated its toxicity against NCI-60, breast cancer (MCF-7), human colon cancer (HCT-116), and HTB-26 in addition to Vero cell lines [80]. The meroterpenoid guanahanolide A consists of a sesterterpene skeleton, which is thought to result from cyclization of geranyl farnesyl diphosphate (GFPP, 2) by a terpene cyclase via a process similar to that described for labdane diterpene biosynthesis [80].

## Clinical trials of marine natural products against cancer

**Fig. 10:**
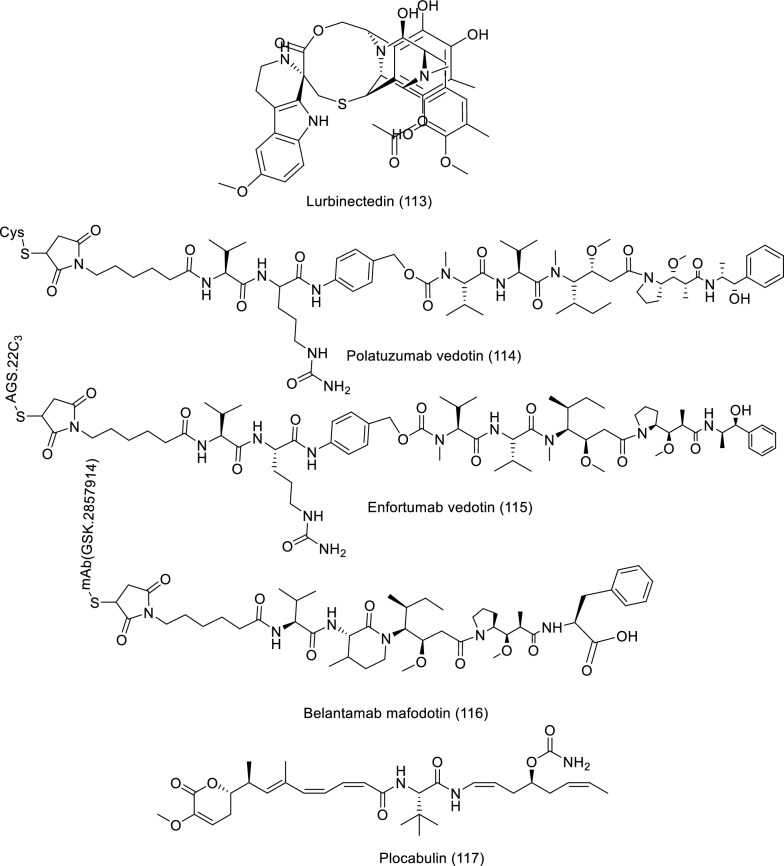
Structures of bioactive compounds derived from marine sources in clinical trials from 2019 to now

To date, several marine organisms' secondary metabolites have been reported with potential uses in the prevention of various cancers. Currently, nine of the 14 marine-derived medications now available on the market are used to treat cancer [16, 81, 82]. Herein we survey the clinical trials of derived bioactive compounds from different marine sources, which were registered or updated from one phase to another one between 2019 to now as shown in Table 8 and Fig. 10.

**Table 8:** Updated list of bioactive compounds derived from marine sources in clinical trials from 2019 to now

Compound Name/	Class of compound	Marine source	Type of cancer	Clinical status/Phase/Study type/	Dose/Route of administration	Mechanism	References
Lurbinectedin (**113**)	Alkaloid	Tunicate	Pancreas cancer	Drug/phase 2/Interventional	3.2 mg/m^2^ (Day 1 of each cycle (one cycle = 3 weeks ± 48 h))/Intravenously	Not reported	https://clinicaltrials.gov, NCT05229588
			Breast cancer		1 mg/vial and 4 mg/vial (Day 1 every three weeks)/Intravenous	The mechanism involves the selective destruction of elongating RNA polymerase II by the ubiquitin/proteasome machinery after its halting on the DNA template	https://clinicaltrials.gov, NCT01525589; [83]
Polatuzumab vedotin (**114**)	Antibody–drug conjugates (anti-CD79b Ab conjugated to MMAE)	Mollusk/Cyanobacterium	Diffuse Large B Cell Lymphoma	Drug/Phase 1 and 2/Interventional	1.8 mg/kg day 2 of cycle 1 and day 1 of successive cycles (each cycle 21 day)/intravenously	Targeting the human B-cell surface antigen CD79b as well as monomethyl auristatin E, which which by binding to tubulin and severing the microtubule network, induces apoptosis while preventing cell division	https://clinicaltrials.gov, NCT04491370; [86]
Enfortumab Vedotin (**115**)	Antibody–drug conjugate (targets Nectin-4 conjugated to MMAE	Mollusk/cyanobacterium	Urothelial Cancer	Drug/Phase 2/Interventional	1.2 mg/kg on days 1, 8, and 15 of each 28-day cycle/intravenously	It has the ability to bind to Nectin-4 expressing cells with high affinity, causing internalization and release of MMAE in target cells, enabling cell-cycle arrest and apoptotic death of Nectin-4 expressing cells	https://clinicaltrials.gov, NCT03219333; [87]
			Metastatic Castration-resistant Prostate Cancer		1.2 mg/kg up to 125 mg (on days 1, 8, and 15 as part of a 28-day cycle)/Not reported	Not reported	https://clinicaltrials.gov, NCT04754191
Belantamab mafodotin (**116**)	Antibody drug conjugate (targeting B-cell maturation antigen conjugated to MMAF	Mollusk/cyanobacterium	Multiple Myeloma	Drug/Phase 2/Interventional	2.5 or 3.4 mg/kg/intravenously	It destroys multiple myeloma cells through triggering apoptosis, boosting antibody-dependent cellular cytotoxicity and phagocytosis, and generating immunogenic cell death,	https://clinicaltrials.gov, NCT03525678; [88]
Plocabulin (PM060184) (**117**)	Polyketide	*Lithoplocamia lithistoides* (Sponge)	Advanced Colorectal Cancer	Drug/Phase 2/Interventional	9.3 mg/m^2^ on Day 1 and Day 8 q^3^wk/intravenously	Not reported	https://clinicaltrials.gov, NCT03427268

Lurbinectedin, an alkaloid analogue of trabectedin, was isolated from tunicate and assessed as a drug (phase 2) for fighting pancreatic cancer with the last update (August 10, 2022) and breast cancer (September 25, 2020). The antitumor activity of lurbinectedin is attributed to the inclusion of a tetrahydroβ‐carboline in their skeleton. The dose of (3.2 mg/m^2^ at day 1 of every cycle) was administered orally for the pancreatic cancer patients, but for breast cancer, the dose is (1 mg/vial and 4 mg/vial at day 1 every three weeks). This clinical study involved 19 participants with ages of 18 and older for pancreatic cancer and 111 participants with ages 18 to 75. The path of action of lurbinectedin was explained by the unalterable stalling of elongating RNA polymerase II on the DNA template and its specific breakdown by the ubiquitin/proteasome workings (https://clinicaltrials.gov, NCT05229588, NCT01525589, [83, 84]). Another example, Polatuzumab vedotin, is an antibody–drug conjugate isolated from mollusk/cyanobacterium involving a monoclonal anti-CD79b coupled to monomethyl auristatin E, which is an anti-mitotic cytotoxic agent. The polatuzumab vedotin was described as an interventional drug in phases 1 and 2 for treating diffuse large B cell lymphoma with last inform (November 3, 2021). It can be administered intravenously with a dosage (of 1.8 mg/kg on day 2 of cycle 1 and day 1 of succeeding cycles). The ages of the 20 participants in the study ranged from 12 to 70 years. Polatuzumab vedotin's mechanism of action was mainly by targeting the human B-cell surface antigen CD79b and monomethyl auristatin E, which blocks cell division and encourages apoptosis by attaching to tubulin and disabling the microtubule network (https://clinicaltrials.gov, NCT04491370, [85, 86]). Belantamab mafodotin is an antibody–drug conjugate isolated from mollusk/cyanobacterium that targets Nectin-4 conjugated to MMAE. The belantamab mafodotin was passed to phase 2 and examined for treating urothelial cancer and metastatic castration-resistant prostate cancer with the last update (June 15, 2022) and (July 3, 2023), respectively. According to urothelial cancer, the participants received a dosage of 1.2 mg/kg on days 1, 8, and 15 of each 28-day cycle intravenously (IV), while metastatic castration-resistant prostate cancer received a dosage of 1.2 mg/kg up to 125 mg intravenously (IV) on days 1, 8, and 15 as part of a 28-day cycle. Belantamab mafodotin's mechanism of action for urothelial cancer was explained by the cell-cycle arrest and apoptosis of Nectin-4-expressing cells (https://clinicaltrials.gov, NCT03219333, NCT04754191, [87]).

## Marketed marine drugs

The anticancer sea-derived medications currently available on the pharmaceutical markets in the EU and/or the USA [22] are shown in Table 9.

**Table 9:** Recent marine anticancer drugs allowed by the EMA and/or the FDA

Generic name	Brand name	Source	Chemical class	Clinical use	CAS No	Marketing authorization date
Lurbinectedin	Zepzelca	Tunicate	Alkaloid	Ovarian cancer	497871-47-3	2020 FDA
Polatuzumab vedotin	Polivy	Mollusk/cyanobacterium	Antibody drug conjugate	Breast cancer	1313206-42-6	2019 FDA 2020 EMA
Enfortumab vedotin	Padcev	Mollusk/cyanobacterium	Antibody drug conjugate	Urothelial cancer	1346452-25-2	2019 FDA 2021 EMA
Belantamab mafodotin	Blenrep	Mollusk/cyanobacterium	Antibody drug conjugate	Multiple myeloma	2050232-20-5	2020 FDA 2020 EMA

## Conclusions and future perspectives

The current review is an updated and expanded version of our prior review, which was published in this journal in 2019. For the current approved anticancer therapeutic agents isolated and identified from marine sources, the time period has been prolonged to include the last five years. According to WHO recent statistics, the cancer burden continues to rise globally. In this contemporary epoch, discovering novel therapeutic structures without side effects to combat lethal diseases, including cancer, is a crucial issue for scientific scholars and governments. Occasionally, preventing, treating, and rehabilitating strategies in cancer cases require a budget that is as high as the one spent in a war. Thus, seeking potential safe anticancer drugs has become an urgent demand. The oceans and seas cover almost 75% of the earth and thus are rich in secondary metabolites with various pharmacological targets. According to our literature survey we have found that, soft corals, sponges then fungi are the primary sources of anticancer drugs identified from marine sources. Terpenoids and alkaloids are the principal chemical classes of these drugs. It was found that these marine structures demonstrate potent preclinical anticancer and cytotoxic activities toward wide ranges of cell lines among them MCF-7, HeLa, PC, L1210 murine leukemia cells, NCI-H460, SW480, HepG2, K562, ASPC-1, H69AR, H69, MDA-MB-231, CRC, brain cancer, ovarian cancer, Ehrlich carcinoma and cervical cancer nonmalignant cell lines (H9c2 and HEK293T), esophageal cancer cell lines (EC109, EC9706, KYSE30, KYSE70, and KYSE450), and human hepatocellular carcinoma SK-Hep-1 compared to common positive control. Taxol, cyclophosphamide, cisplatin, and doxorubicin are some of the examples combating cancers via different mechanisms, including inducing cell growth inhibition and apoptosis via ROS production and the RXRα-mediated PI3K/Akt signaling pathway, suppressing the MAPK/ERK pathway and modulating the extrinsic pathway, activation of the p38 MAPK pathway and p38 phosphorylation in tumor tissue, inhibiting VEGF, MCP-1, RANTES expressions and angiogenesis, induced changes in the phosphorylation status and altered expression of HSP60, HSP70 and PRAS40 proteins, regulation of Bax expression, which is a biomarker of tumor growth and regulating NF-κB signaling pathways. Lately, six marine structures have been clinically approved as anticancer medications, among them 4 compounds, namely, Lurbinectedin, Polatuzumab vedotin, Enfortumab vedotin, and Belantamab mafodotin have been authorized by the FDA and/or EMA as anticancer drugs against ovarian cancer, breast cancer, urothelial cancer, and multiple myeloma, respectively. Taken together, it is nowadays evident that marine products are crucial for supplying a platform for several approved anticancer drugs.

## Data Availability

No data was used for the research described in the article.
